# TR3 Enhances AR Variant Production and Transactivation, Promoting Androgen Independence of Prostate Cancer Cells

**DOI:** 10.3390/cancers14081911

**Published:** 2022-04-10

**Authors:** Tuyen Thanh Tran, Keesook Lee

**Affiliations:** Laboratory of Developmental Genetics, School of Biological Sciences and Technology, Chonnam National University, Gwangju 61186, Korea; thanhtuyentran2812@gmail.com

**Keywords:** TR3, androgen receptor, AR splicing variants, androgen-independent activity, castration-resistant prostate cancer

## Abstract

**Simple Summary:**

Advanced prostate cancer development is associated with androgen-independent AR signaling. TR3 overexpression alters AR expression, splicing process, and transactivation towards increasing the androgen independence of AR signaling in prostate cancer cells. These results suggest that TR3 is a pivotal factor in the progression of prostate cancer to advanced form.

**Abstract:**

The pro-oncogenic function of TR3, an orphan nuclear receptor, has been reported in prostate cancer. However, the roles of TR3 in androgen receptor (AR) expression and signaling in prostate cancer cells are poorly understood. Database analysis revealed that TR3 expression level is elevated in prostate tumors, and is positively, although weakly, correlated with that of AR. TR3 overexpression increased the production of AR splice variants in addition to general upregulation of AR expression. TR3 interacted with some spliceosomal complex components and AR precursor mRNA, altering the splice junction rates between exons. TR3 also enhanced androgen-independent AR function. Furthermore, TR3 overexpression increased cell proliferation and mobility of AR-positive prostate cancer cells and stimulated tumorigenesis of androgen-independent prostate cancer cells in mouse xenograft models. This is the first study to report that TR3 is a multifunctional regulator of AR signaling in prostate cancer cells. TR3 alters AR expression, splicing process, and activity in prostate cancer cells, increasing the androgen independence of AR signaling. Therefore, TR3 may play a crucial role in the progression of prostate cancer to an advanced castration-resistant form.

## 1. Introduction

Prostate cancer is one of the leading lethal malignancies in males, and androgen receptor (AR), a ligand-dependent transcription factor, plays a pivotal role in the development, progression, and metastasis of prostate cancers [1]. AR has three main functional domains: NH2-terminal transactivation domain (NTD), DNA-binding domain, and ligand-binding domain (LBD). Alterations in AR signaling, which include AR amplification and mutations that lead to increased AR activity and AR activation by an outlaw pathway in a ligand-independent manner, are important mechanisms that drive prostate cancer survival and progression to castration-resistant prostate cancer (CRPC) [2]. Recent reports have also revealed that increased expression of constitutively active AR variants (AR-Vs), which lack LBD, is associated with the development of advanced CRPC, leading to the failure of hormone therapy [3]. Among the multiple AR-Vs reported, AR-V7 (AR3) is the most common in prostate cancer cell lines and tumors and is generated by splicing between exon 3 and cryptic exon 3 (CE3) as well as exon 3 duplication [4]. Mutations and genomic rearrangements are the two major causes of multiple drug resistance during prostate cancer progression, especially during the development of advanced CRPCs [5,6]. These mutations and genomic rearrangements result in the generation of alternative promoters and cryptic splice sites that drive an alternative splicing process.

Several splicing factors have been found to be associated with alternative splicing events in the AR precursor mRNA (pre-mRNA) in prostate cancer cells. Among these, heterogeneous nuclear ribonucleoprotein (hnRNP) A2B1, hnRNP E1/E2, and HUR (ELAV-like protein 1, ELAVL1) are RNA-binding proteins (RBPs) that bind to AR pre-mRNA and regulate the expression of AR-Vs in advanced CRPCs [7,8]. hnRNP E1/E2 contains KH domains and HUR contains RRM domains, and both of these domain types bind to the UC-rich region in AR pre-mRNA [7]. In contrast, hnRNP A/B proteins, especially hnRNP A1 and A2B1, which contain RRM domains, bind to AR pre-mRNA splice sites, and enhance the expression of some AR-Vs, particularly AR-V7, AR-V1, AR-V4, and AR-V5, in CRPCs [8]. Enrichment of hnRNP A1/A2B1 at splice sites involved in AR-V production was further increased in enzalutamide-resistant prostate cancer cells [8]. Additionally, NOVA-2 is an alternative splicing factor, whose expression is upregulated in some cancers [9], although its role in prostate cancer is poorly understood. It is a KH domain-containing RBP, which binds to the YCAY sequence in RNA and regulates the stabilization and transport of intron-excised RNA.

Human orphan nuclear receptor TR3 (also known as NR4 A1) is an immediate early response gene induced by a diverse range of signals, including stressors, cytokines, growth factors, and small molecular compounds. TR3 expression is associated with many physiological and pathological processes, such as cell survival and death, inflammation, and cancer development [10]. TR3 is upregulated in many cancers, including lung, colorectal, breast, and prostate cancers [11]. In addition, TR3 signaling activation enhances cancer cell proliferation and tumor progression [11], while loss of TR3 function by retinoid and its derivative compounds induces apoptosis [12]. The modification of TR3 protein has been reported in regard to its biological functions [13,14]. For example, the phosphorylation of TR3, such as pS351-TR3 via PI3K/AKT signaling pathway and pS355-TR3 via MAPK signaling pathway, activates TR3 activity to inhibit cell pro-apoptosis [13,14]. Treatment of TGF-*β*, which induces cell invasion as well as epithelial-mesenchymal-transition in many cancers, promotes the phosphorylation and nuclear export of TR3 to inhibit pro-apoptosis in cancer cells, while TR3 antagonists block TGF-*β*-induced nuclear export of TR3 [13]. TR3 overexpression increases prostate cancer cell viability [11]. However, the roles of TR3, especially in AR expression and signaling, are poorly understood.

The present study is the first to demonstrate that TR3 affects the AR splicing process as well as its expression and further enhances androgen-independent AR activity in prostate cancer cells. These results, together with the positive, although weak, correlation between TR3 and AR expression levels in prostate tumors and the stimulation of tumorigenesis by TR3 overexpression, suggest that TR3 is a pivotal controller of AR signaling in prostate cancer cells and plays a crucial role in cancer progression and the maintenance of advanced CRPCs.

## 2. Materials and Methods

### 2.1. Cell Lines

LNCaP (CRL-1740), HEK 293T (CRL-11268), PC-3 (CRL-1435), PPC-1 (HTB-190) and DU145 (HTB-81) cells were purchased from the American Type Culture Collection (ATCC; Manassa, VA, USA). These ATCC cell lines were authenticated by STR analysis. C4-2 and CWR22Rv1 cells were obtained from Dr. Leland W. Chung’s laboratory (Cedars-Sinai Medical Center, Los Angeles, CA, USA), which were characterized by in vitro characterization techniques [15,16]. A recombinant adenovirus E1 expressing HEK-293 (AD-293; 240085) cells were purchased from Agilent Technologies, Inc. (Santa Clara, CA, USA). LNCaP, C4-2, CWR22Rv1, and PC-3 cells were maintained in RMPI-1640 media (HyClone, Logan, UT, USA) with 5% fetal bovine serum (FBS) (Gibco, Grand Island, NY, USA). PPC-1, AD-293, and HEK 293T cells were cultured in DMEM (HyClone, USA) with 10% FBS. Media containing 5% charcoal-stripped serum (cFBS) was used for the starvation and steroid studies. All cell lines were maintained in the presence of 100 units/mL penicillin and 100 mg/mL streptomycin (Gibco, USA) in a 5% CO_2_ incubator at 37 °C.

### 2.2. Generation of Inducible TR3-Overexpressing CWR22Rv1 Cells

FLAG-TR3 was excised from pcDNA3.FLAG-TR3 using *EcoR*V and *Hind*III, blunted, and inserted into pBI-EGFP at the *Nhe*I site to generate pBI-EGFP-FLAG-TR3. CWR22Rv1 cells were transiently co-transfected with pUHDrtTA2S-M2 (TRE) (Dr. H. Bujard; ZMBH, Heidelberg, Germany) and pBI-EGFP-FLAG-TR3 or pBI-EGFP (EV; Clontech, Palo Alto, CA, USA) and then selected using 500 ng mL^−1^ G418 (Geneticin; Invitrogen, Carlsbad, CA, US). The selected subclones were treated with 2 µg mL^−1^ doxycycline (Sigma, St. Louis, MO, USA). TR3 overexpression was confirmed by RT-PCR analysis.

### 2.3. Next-Generation Sequencing (NGS) High Throughput RNA-Seq Analysis

CWR22Rv1 cells were infected with AdTR3 or AdCtrl, maintained for 24 h, and then harvested for RNA isolation followed by whole transcriptome analysis (Ebiogen Inc., Seoul, Korea). Whole transcriptome analysis was performed once with the cell population infected with TR3-overexpressing adenovirus, which contained cells expressing different levels of TR3. RNA was converted to cDNA using a Clontech SMARTer Stranded RNA-Seq Kit with the proprietary SMART stranded N6 primer and SMARTScribeTM reverse transcriptase. The postcapture sequencing libraries were pooled and sequenced on the Illumina HiSeq 2500 platform using the 2 × 100 bp settings. Reads were mapped to the human genome reference HG19 using MapSplice. Gene expression was quantified using EdgeR and quantile normalized. Gene set enrichment analysis was performed using GSEA program and DAVID analysis on a preranked list of differentially expressed (>1.5-fold) genes between AdTR3- and Ad-Ctrl-infected CWR22Rv1 cells. Genes were clustered, and the difference in gene expression was classified using the MeV program. Novel splice variants were detected by aligning the novel splices against whole transcriptomes. Alterations in the splice junction rates between exons in the full-length AR gene were analyzed through Integrative Genomic Viewer (IGV) analysis.

### 2.4. Single-Strand RNA (ssRNA) Protection Assays

Thirty-mer ssRNA labeled with biotin at the 5′ end (ssRNA oligo) was designed for RNA-protein interaction assays. ssRNA oligo sequence was identical to that found within Reg4, which contains one hnRNP A2B1 binding site overlapping with one HUB/HUR and two other HUB binding sites but has no hnRNP E1/E2 binding site. ssRNA oligo was incubated with or without the purified protein (FLAG-TR3 or splicing factors hnRNP A2B1 and HUB/HUR) in a binding buffer [20 mM Tris (pH 8.0), 0.5% sucrose, 1 mM DTT, 1 mM MgCl_2_, 1 mM EDTA, and 2% glycerol in DEPC water] overnight at 4 °C. RNA and protein degradation was minimized by adding RNase (RNasin) and protease inhibitors, respectively. Reactions were analyzed on a 3% horizontal agarose gel in TBE buffer containing 10 µg/mL EtBr. Gels were cooled and run in cooled TBE buffer. Free RNA and RNA-protein complex signals were detected using a UV transilluminator. ssRNA sequence is listed in Table S1.

### 2.5. Cross-Linked RNA-Immunoprecipitation (CLIP)

CWR22Rv1 cells were infected with AdTR3 or AdCtrl, maintained for 24 h, and cross-linked with 1% formaldehyde (Sigma-Aldrich, St. Louis, MO, USA) for 10 min. The fixed cells were then subjected to cross-linked RNA-immunoprecipitation (CLIP) analysis as previously described [17] with minor modifications. The protein-bound primary RNA fragments were enriched through immunoprecipitation with anti-hnRNP A2B1 antibodies (Table S2). DNA contaminants were removed by treating with RNase-free DNase (ThermoFisher Scientific, Rockford, IL, USA) at 37 °C for 30 min, following which, RNA fragments were obtained using chloroform extraction, and cDNA was synthesized using random hexamers (Enzynomics) and M-MLV Reverse transcriptase kit (Promega, Madison, WI, USA). Enrichment of AR pre-RNA fragments was analyzed via RT-PCR using the CLIP primers listed in Table S1.

### 2.6. ssRNA Oligo Pull-Down Assays

The interactions between ssRNA oligo and proteins (hnRNP A2B1, hnRNP E1/E2, HUB/HUR, and TR3) were examined using ssRNA oligo pull-down assays, as previously described [4], but with minor modifications. ssRNA oligo was immobilized onto AccuNanoBead^TM^ Streptavidin Magnetic Nanobeads (400 nm, 5 mg/mL; Bioneer, Korea) for 1 h at 4 °C in the presence of RNasin, brewer’s yeast tRNA (Roche, Basel, Switzerland), and salmon sperm-sheared DNA (ssDNA). Purified TR3 and splicing factor proteins were added into each interaction reaction in the presence of protease inhibitors and BSA. The interaction reactions were incubated overnight at 4 °C. ssRNA oligo-bound proteins were analyzed using western blot analysis. RNA pull-down assay was also performed using whole cell lysates extracted from CWR22Rv1 cells infected with AdTR3 or AdCtrl. Total proteins from each cell lysate were used in each binding reaction.

### 2.7. Xenograft Animal Model

Healthy and microbiologically monitored 4-week-old male NOD.CB17-Prkdc^SCID/J^ mice, obtained from Korea Research Institute of Bioscience and Biotechnology (Daejeon, Korea), were gently intraperitoneally injected (i.p.) with 100 µL of injectable anesthetics [Zoletil 50: Rompun: saline buffer (20:10:270)]. Mice were then warmed on a veterinary warming system. Inducible TR3-overexpressing (TR3; subline #2) or control (EV) CWR22Rv1 cell clones (2 × 10^6^ cells/site mixed 1:1 with Matrigel) were subcutaneously injected (s.c.) into the shoulders of each mouse. The following day, mice were provided distilled water with or without 2 µg/mL doxycycline. Tumor size and volume were monitored and measured three times a week. Two days after the last tumor measurement, the animals were subjected to CO_2_-induced euthanasia and sacrificed, and the tumors were extracted and weighed. Animals were maintained inside a cleaned bench in an animal room with a 12-h light/dark cycle and controlled temperature during all processes. The sterilized mouse cages, water, food, and bedding used for the animal study were replaced once a week. Ultra-fine II short needles (U-100 INSULIN 30 gauge 5/16′’ (8 mm) needle) were used for all injections to minimize any pain and lesions. Tumor volumes were calculated using the following formula: length × width × height × 0.5236 (V = π × L × W × H/6 = L × W × H × 0.5236) [18]. Statistical significance was calculated using two-tailed *t*-test analysis. *p*-value < 0.05 was considered statistically significant. All animal procedures were approved by the Institutional Animal Care and Use Committee (IACUC) of Chonnam National University (permit number: 2012-44). Animal experiments have been performed in accordance with the ARRIVE/NC3R guidelines.

### 2.8. Quantification and Statistical Analysis

Animal experiments were performed using six mice in each group. Data, which were obtained from more than three independent experiments, are presented as the mean ± SEM. Statistical significance was calculated using one-way ANOVA with Tukey’s post-hoc test, two-way ANOVA with Bonferroni’s post hoc test, and two-tailed *t*-test analysis. *p*-value < 0.05 was considered statistically significant.

## 3. Results

### 3.1. TR3 Regulates the Expression of AR and AR-Vs in Prostate Cancer

**Figure 1:**
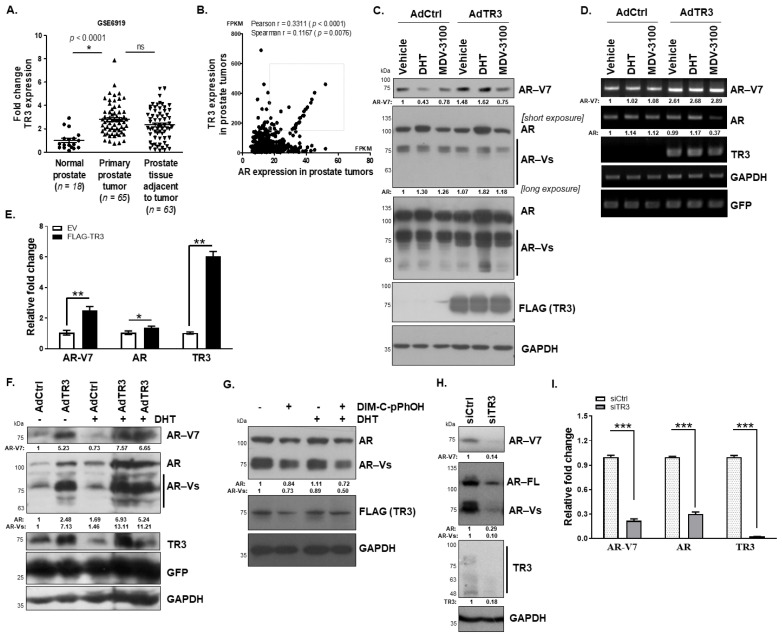
TR3 regulates the expression of AR and AR-Vs in prostate cancer. (**A**) Elevation of TR3 in prostate tumors. The graph shows TR3 mRNA levels in normal human prostate (*n* = 18), primary prostate tumor (*n* = 65), and prostate tissue adjacent to tumor (*n* = 63). The data were extracted from the public microarray data established by the Pathology Department of Houston Methodist University Hospital. The fold change of TR3 expression in tissues was obtained by normalizing TR3 level in each tissue to the mean of those in normal prostate tissues. Data are shown as mean ± SEM. *, *p* < 0.001; ns, not significant; one-way ANOVA analysis with Tukey’s post-hoc test. (**B**) Positive correlation between TR3 and AR expression levels in prostate tumors. The x and y axes denote Fragments Per Kilobase of exon per Million reads (FPKM), which were extracted from The Cancer genome Atlas (TCGA) Prostate Tumors’ Pathology Database (*n* = 494). Pearson r or Spearman r and *p* values were analyzed using correlation analysis with two-tailed Pearson or two-tailed Spearman test, respectively. (**C**–**F**) TR3 overexpression increases the expression levels of AR and AR-Vs. Representative western blot (**C**) and RT-PCR (**D**) analysis showing the protein levels of AR and AR-Vs, and the appearance of other AR-Vs in AdTR3- or AdCtrl-infected CWR22Rv1 cells, which were treated with 10 nM DHT, 10 µM MDV-3100, or vehicle for 24 h. GFP was used as a control for the efficiency of adenovirus infection. Quantitative RT-PCR (qPCR) analysis showed alterations in the level of AR mRNAs, which contain exon 1–exon 2 (AR) and exon 3–CE3 (AR-V7) in CWR22Rv1 cells transfected with FLAG-TR3 or empty vector (EV) (**E**). (**F**) Representative western analysis showing the protein levels of AR and AR-Vs, especially AR-V7, in CWR22Rv1 cells infected with AdTR3 or AdCtrl in the absence or presence of 10 nM DHT for 3 days. (**G**–**I**) Inhibition of TR3 function or silencing of TR3 expression significantly decreases the expression levels of AR and AR-Vs. Representative western blot analysis showing AR, AR-V, and TR3 protein levels in CWR22Rv1 cells that were overexpressed with TR3 and treated with 20 µM of TR3-specific antagonist (DIM-C-pPhOH) in the presence or absence of 10 nM DHT for 24 h (**G**). Representative western blot analysis showing AR and AR-V protein levels in CWR22Rv1 cells silenced with siTR3 for 3 days (**H**). qPCR analysis showing AR, AR-Vs, and TR3 mRNA levels in CWR22Rv1 cells transfected with siTR3 or siCtrl (**I**). Data are shown as mean ± SEM. *, *p* < 0.05; **, *p* < 0.01; ***, *p* < 0.001; one-way ANOVA analysis with Tukey’s post-hoc test. Values below western blot blots and gels indicate the relative band intensity of each gene normalized to GAPDH. All uncropped western blot and gel original images are shown in Figure S7.

To investigate the oncogenic function of TR3 in prostate cancer, we analyzed its expression profile in prostate tumors (Gene Expression Omnibus database) and evaluated the correlation between TR3 and AR expression levels in prostate tumor patients (The Cancer Genome Atlas database). We found that TR3 expression is upregulated in primary prostate tumors (Figure 1A) and that its expression level is positively, although weakly, correlated with that of AR (Figure 1B). Interestingly, there is a group of patients with a clear linear relationship between AR and TR3 expression, in which most of the patients are 60–75 year-old white men, who were disease-diagnosed over 2 years (2–6 years). Intriguingly, equally high levels of TR3 expression were observed in normal prostate tissues adjacent to primary tumors (Figure 1A). This is probably because immune deficiency and alteration of the tumor microenvironment by TR3 affects the physiology of tumor-adjacent cells, facilitating cancer progression [19]. In addition, TR3 expression was detected in all tested AR-positive prostate cancer cell lines in spite of a very low basal level in LNCaP cell line, but not in AR-negative prostate cancer cell lines and was induced by 5*α*-dihydrotestosterone (DHT) (Figure S1A), indicating TR3 as an androgen-responsive gene.

As TR3 and AR expression levels are positively correlated in prostate tumors, we investigated whether TR3 regulates AR expression. Overexpression of TR3 enhanced the protein levels of AR-Vs including AR-V7 in prostate cancer cells which were treated with DHT (Figure 1C and Figure S1B). We also observed other AR-Vs in TR3-overexpressing cells, which were hardly detected in the controls (Figure 1C, long exposure; Figure S1B, long exposure). In addition, the mRNA levels of AR and AR-V7 were enhanced in these TR3-overexpressing cells treated with or without androgen, when examined using primers specifically covering exon 3/exon 4 (AR) and exon 3/CE3 (AR-V7) (Figure 1D and Figure S1C). Furthermore, TR3 overexpression increased the mRNA level of AR-V7 significantly more than that of the exon 1/exon 2-containing AR in CWR22Rv1 cells (Figure 1E), which was quantified through qPCR, pointing to the effects of TR3 on AR pre-mRNA splicing process as well as AR transcription. Intriguingly, long-term overexpression of TR3 for 3 days increased the protein level of AR-V7 significantly more than that of full-length AR in CWR22Rv1 cells as well as in other prostate cancer LNCaP and C4-2 cells (Figure 1F and Figure S1D). In addition, treatment with the TR3 antagonist, DIM-C-pPhOH, strongly decreased the protein level of AR and AR-Vs in the presence or absence of DHT (Figure 1G and Figure S1E). As expected, silencing TR3 decreased the protein and mRNA levels of AR and AR-Vs in AR-positive prostate cancer cells (Figure 1H,I and Figure S1F). However, in TR3-knockdown CWR22Rv1 cells, the decreased AR and AR-V protein levels by TR3 silencing were not restored following treatment with MG-132 and chloroquine, which are blockers of protein degradation pathways (Figure S1G), suggesting that TR3 regulates AR expression at mRNA level.

### 3.2. TR3 Regulates the Expression of AR Splicing Variants, Altering Splice Junction Rates between Exons

**Figure 2:**
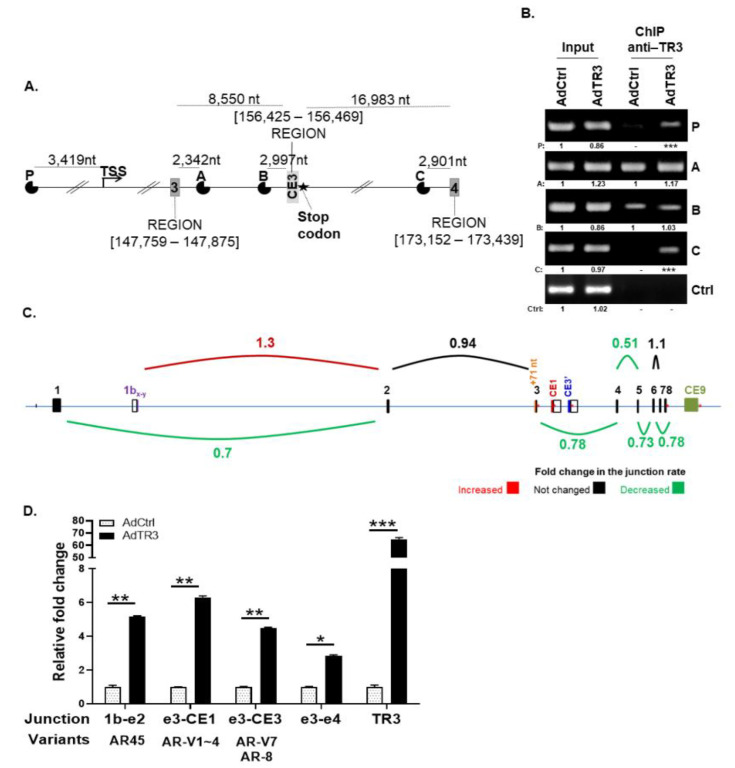
TR3 regulates the expression of AR splicing variants, altering splice junction rates between exons. (**A**,**B**) TR3 binds to the promoter and intron regions of AR gene. Schematic presentation of putative TR3 binding sites (P, A, B, and C) in AR gene (**A**). Recruitment of TR3 protein to putative TR3 binding sites within the promoter and intron regions of the AR gene was determined via ChIP assays using anti-TR3 antibody (**B**). Values below representative gels indicate the intensity ratio of AdTR3-infected sample relative to AdCtrl-infected sample in each region. *** indicates the fold increase >5. CWR22Rv1 cells were infected with AdTR3 or AdCtrl. Changes in TR3 enrichment at putative TR3 binding sites was examined using PCR. The loading control (Ctrl) was *β*-ACTIN. (**C**,**D**) TR3 overexpression alters the splice junction rate between exons of AR pre-mRNA. CWR22Rv1 cells were infected with AdTR3 or AdCtrl, and the samples were analyzed using next-generation sequencing (NGS) high-throughput RNA-Seq analysis. Splice junction rate between exons within the pre-mRNA of AR-FL was analyzed using Integrative Genomic Viewer (IGV) analysis (**C**). qPCR analysis showed alterations of the splice junction rate between AR-V exons (**D**). Data are shown as mean ± SEM. *, *p* < 0.05; **, *p* < 0.01; ***, *p* < 0.001; one-way ANOVA analysis with Tukey’s post-hoc test. All uncropped gel original images are shown in Figure S7.

The coupling of transcription and splicing processes is well-known [20,21,22]. Therefore, to further investigate the effects of TR3 on AR splicing process and expression, we first analyzed the potential binding sites of TR3 (TRE: AGGTCA) ~5 kb upstream of the transcription start site and in the region covering the whole AR gene using Annhyb, a bioinformatics-based tool for sequence analysis. Four potential binding sites (P, A, B, and C) were found; site P was located ~3.4 kb upstream of the transcription start site, while the other three sites (A–C) were located within AR gene introns. Site A was located near the 3′ splicing site (3′ss) of exon 3, site B near the 5′ splicing site (5′ss) of CE3, and site C near the 5′ss of exon 4 (Figure 2A). ChIP assays showed that TR3 might bind to all four sites; however, TR3 overexpression preferably enhanced TR3 binding to the regions containing sites P and C, but not sites A and B (Figure 2B). These results suggest that TR3 binds to TREs in the AR gene and regulates its expression. However, we cannot rule out the possibility that TR3 binding to intron regions, especially to site C, affects the AR pre-mRNA splicing process.

To investigate the alteration of AR splicing events in TR3-overexpressing prostate cancer cells, we performed next-generation sequencing (NGS) high-throughput RNA-Seq analysis of RNA obtained from TR3-overexpressing and control CWR22Rv1 cells. The INDEL coupling with Integrative Genomic Viewer (IGV) analysis of RNA-Seq data of AR transcripts revealed that splice junction rates between exons within the pre-mRNA of full-length AR gene were generally decreased (up to 50%) when TR3 was overexpressed in CWR22Rv1 cells, while the splice junction rate between exon 1b_y_ and exon 2 increased (~30%) (Figure 2C). The splicing between exon 1b_y_ and exon 2 has been identified in AR transcript variant 2 (also known as AR45), a short transcript that lacks exon 1, which is produced by utilizing an alternative transcription start site located between −3 and +5 of exon 1b_y_ [23,24]. Overexpression of AR45 may either repress or stimulate AR transactivation, depending on the cellular context.

We then validated the expression of AR splicing variants by performing qPCR analysis using specific primer pairs covering exon 1b_y_, exon 2, exon 3, cryptic exon CE1, cryptic exon CE3, and exon 4 (Figure 2D). The results showed that the increase in the level of RNA containing splice junctions between exon 1b_y_ and exon 2 (found in AR45), exon 3 and CE1 (found in AR-V1~4), and exon 3 and CE3 (found in AR-V7 and AR-8) was higher than the general increase in total AR mRNA containing the exon 3–exon 4 junction, which was upregulated by TR3 activation of the AR promoter (Figure 2D). Overexpression of TR3 also markedly increased the levels of RNA containing exon 1b_y_–exon 2 and exon 3–CE1 in C4-2 cells (Figure S2). AR-Vs containing exon 3–CE1 or exon 3–CE3 were hardly detectable in LNCaP cells when TR3 is overexpressed only for 24 h (Figure S2), although 3 day-overexpression increased the protein level of AR-V7 (Figure S1E). These results suggest that TR3 affects the AR splicing process, especially in advanced CRPC cells, such as CWR22Rv1 and C4-2 cells.

### 3.3. TR3 Physically Interacts with Some Splicing Factors Involved in AR Splicing Process

**Figure 3:**
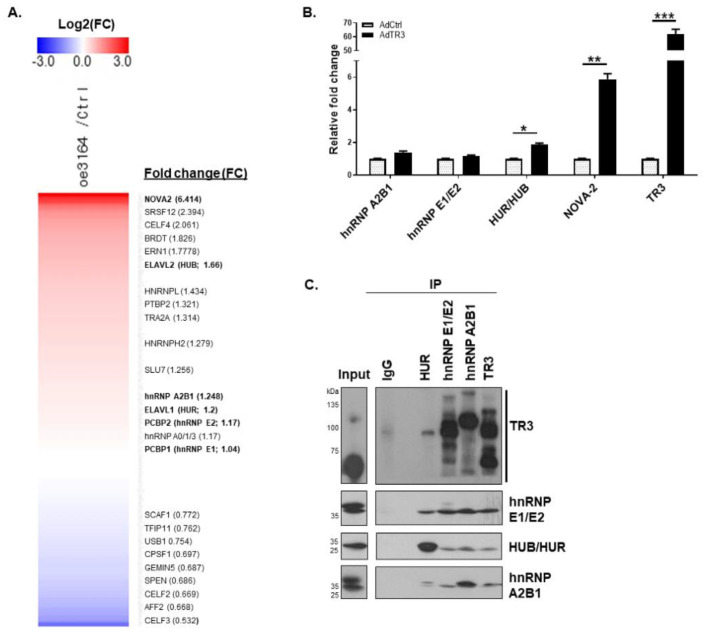
TR3 physically interacts with some splicing factors involved in the AR splicing process. (**A**,**B**) TR3 overexpression alters the expression level of some factors involved in the splicing of AR pre-mRNA. Heatmap of RNA–seq data showing alterations in the expression level of some splicing factors in TR3-overexpressing CWR22Rv1 cells (**A**). qPCR analysis presenting mRNA levels of several splicing factors (hnRNP A2B1, hnRNP E1/E2, HUB/HUR, and NOVA-2) in CWR22Rv1 cells infected with AdTR3 or AdCtrl (**B**). Data are shown as mean ± SEM. *, *p* < 0.05; **, *p* < 0.01; ***, *p* < 0.001; one-way ANOVA analysis with Tukey’s post-hoc test. (**C**) TR3 physically interacts with splicing factors involved in the splicing of AR pre-mRNA. Representative immunoprecipitation (IP) analysis showed physical interactions between TR3 and several splicing factors (hnRNP A2B1, hnRNP E1/E2, and HUB/HUR) in CWR22Rv1 cells infected with AdTR3. Immunoprecipitation was performed using anti-TR3, anti-hnRNP A2B1, anti-hnRNP E1/E2, and anti-HUR antibodies. Protein levels were analyzed via western blot analysis using anti-TR3, anti-hnRNP A2B1, anti-hnRNP E1/E2, and anti-HUR antibodies. All uncropped western blot original images are shown in Figure S7.

Because splicing factors and spliceosomal complexes play a crucial role in the regulation of the splicing process, we first examined whether TR3 overexpression altered the expression of some splicing factors, particularly NOVA-2, HUB (ELAV-like protein 2, ELAVL2), and other splicing factors involved in alternative splicing of AR pre-mRNA, such as hnRNP A2B1, hnRNP E1/E2, and HUR. RNA-Seq analysis revealed ~6.4-fold induction of NOVA-2 and ~1.7-fold induction of HUB in TR3-overexpressing cells compared with the control (Figure 3A). The increase in NOVA-2 and HUB mRNA levels was confirmed through qPCR and RT-PCR analysis (Figure 3B and Figure S3A). Western blot analysis revealed that the NOVA-2 protein level was also increased to some extent (Figure S3B). Other splicing factors, such as hnRNP A2B1 and hnRNP E1/E2, were expressed at high basal levels in CWR22Rv1 cells and showed no significant changes in their mRNA and protein levels upon TR3 overexpression (Figure S3).

hnRNP A2B1 plays a critical role in the control of the AR splicing process [8], and it likely interacts with TR3, based on the analysis of the protein interaction network [25]. Because the protein–protein interaction between TR3 and splicing factors might alter spliceosomal component recruitment and complex formation to control the AR splicing process, we investigated the physical interactions between TR3 and splicing factors (hnRNP A2B1, HUB/HUR, and hnRNP E1/E2) in TR3-overexpressing CWR22Rv1 cells. Co-immunoprecipitation assays revealed that TR3 interacted with splicing factors hnRNP E1/E2 and hnRNP A2B1 (Figure 3C). Interestingly, different forms of TR3 appeared to interact with different splicing factors. Since modifications of TR3 play a role in protein–protein interaction [13,14], it is possible that some modified forms of TR3 have a privilege or are necessary to interact with certain splicing factors. These results suggest that TR3 may be associated with spliceosomal complexes on AR pre-mRNA and may alter the splicing process.

### 3.4. TR3 Overexpression Alters the Recruitment of Some Splicing Factors near Splicing Sites, Interacting with AR Pre-mRNA

**Figure 4:**
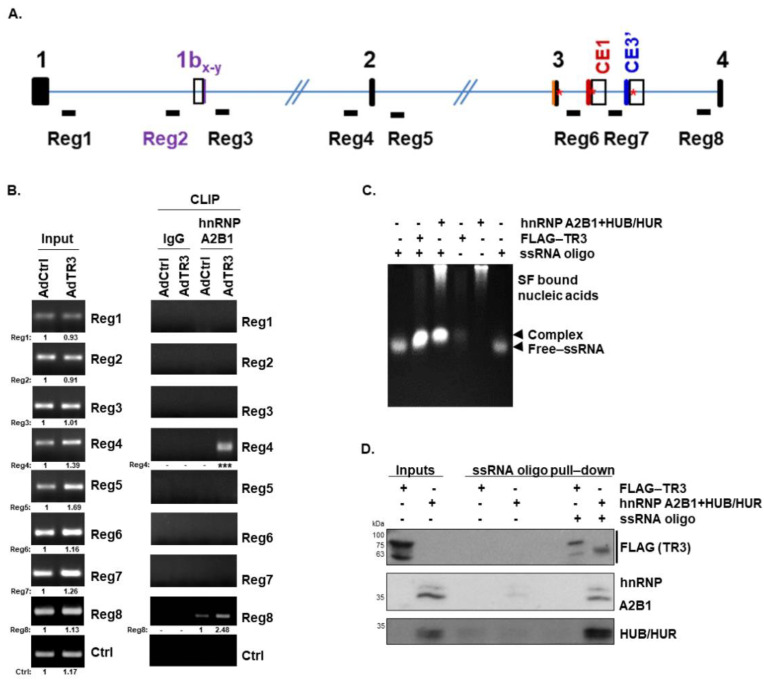
TR3 overexpression alters the recruitment of some splicing factors near splicing sites, interacting with AR pre-mRNA. (**A**,**B**) TR3 overexpression alters the recruitment of some splicing factors to AR pre-mRNA. Schematic presentation of intronic regions marked Reg1–8 in AR pre-mRNA, which were selected for CLIP analysis (**A**). CLIP analysis using anti-hnRNP A2B1 antibody showed an enrichment of hnRNP A2B1 at Reg4 and Reg8 within AR pre-mRNA in TR3-overexpressing CWR22Rv1 cells (**B**). Values below gels indicate the intensity ratio of the AdTR3-infected sample relative to AdCtrl-infected sample in each region. *** indicates the fold increase >5. (**C**,**D**) TR3 interacts with AR pre-mRNA. ssRNA protection assays performed using purified TR3 and splicing factors (hnRNP A2B1 and HUB/HUR) showed protection of a 30mer ssRNA oligo of AR-Reg4 labelled with biotin at the 5′ end (ssRNA oligo) (**C**). ssRNA pull-down assays performed using the same purified protein fractions show the binding of TR3 and splicing factors to ssRNA oligo (**D**). Protein levels were analyzed via western blot analysis using anti-FLAG, anti-hnRNP A2B1, and anti-HUR antibodies. All uncropped western blot and gel original images are shown in Figure S7.

We further investigated whether TR3 altered the recruitment of splicing factors near splice sites. We chose 8 regions, designated Reg1–8, located ~1 kb upstream and/or downstream of the exon/intron junction of AR pre-mRNA and covering exons 1–4 (Figure 4A). This is because spliceosomal components, such as snRNPs (U2/U2AF snRNP and U1 snRNP) and other hnRNPs, mostly bind to 3′ss and 5′ss of introns and branch sites [26], and the region covering exons 1–4 contains cryptic exons, which are frequently found in AR-Vs. Putative binding sites of some RBPs (hnRNP A2B1, hnRNP E1/E2, HUB/HUR, and NOVA2) in the 1.17-Mb AR pre-mRNA were investigated and found within Reg1–8, using SpliceAid program (a database of strictly experimentally assessed target RNA sequences in humans). Additionally, cross-linked RNA immunoprecipitation (CLIP) assays using anti-hnRNP A2B1 antibody and TR3-overexpressing or control CWR22Rv1 cells revealed strong enrichment of hnRNP A2B1 at Reg4, which contains several putative hnRNP A2B1 binding sites, upon TR3 overexpression (Figure 4B). We also detected the enrichment of hnRNP A2B1 at Reg8, which contains no putative binding site for hnRNP A2B1, but it has hnRNP E1/E2 and HUB/HUR binding sites. It is possible that hnRNP A2B1 enrichment at Reg8 was due to protein–protein interactions between hnRNP A2B1 and other spliceosomal complex components.

To examine whether TR3 is associated with the spliceosomal complex at Reg4, we performed in vitro RNA-protein interaction assays involving single-strand RNA (ssRNA) protection and ssRNA oligo pull-down assays. A 30-mer ssRNA labeled with biotin at the 5′ end (ssRNA oligo) was designed to include the sequence found within Reg4, which contains one hnRNP A2B1 binding site overlapping with one HUB/HUR and two other HUB binding sites, but it has no hnRNP E1/E2 binding site. ssRNA protection assays, which were performed using the ssRNA oligo and either purified TR3 or splicing factors (hnRNP A2B1 and HUB/HUR), showed that both TR3 and the splicing factors bound to and protected this ssRNA oligo from degradation (Figure 4C). The ssRNA oligo migrated on agarose gel based on the formation of RNA-TR3 or RNA-splicing factor complex. Furthermore, ssRNA oligo pull-down assays performed using the same purified TR3 and splicing factors revealed that TR3 and the splicing factors, hnRNP A2B1 and HUB/HUR, bound to the ssRNA oligo (Figure 4D). Together, these results suggest that TR3 binds to AR pre-mRNA and alters a recruitment of some splicing factors near splicing sites, which is a novel function of TR3.

### 3.5. TR3 Enhances Androgen-Independent and Androgen-Dependent Transactivation of ARs

**Figure 5:**
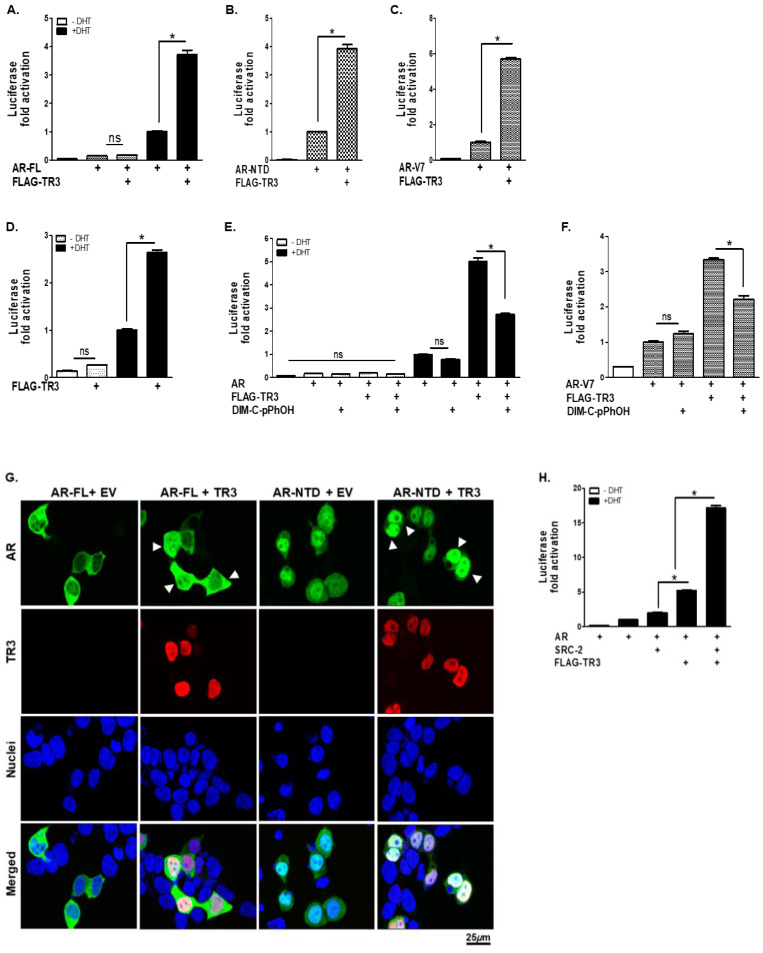
TR3 positively regulates the transactivation of ARs in both androgen-dependent and androgen-independent manners. TR3 enhances the androgen-dependent and -independent transactivation of ARs. (**A**–**D**) TR3 overexpression significantly enhances the transactivation of ARs. Both PPC-1 cells overexpressed with: AR-FL (**A**), AR-NTD (**B**), or AR-V7 (**C**), and LNCaP cells (**D**) were transiently transfected with FLAG-TR3 or empty vector (EV) along with pARE2-TATA-luc and treated with 1 nM DHT or vehicle. (**E**,**F**) TR3-mediated enhancement of the transactivation of ARs was impaired by treatment with TR3 antagonist (DIM-C-pPhOH). PPC-1 cells were co-transfected with AR-FL (**E**) or AR-V7 (**F**) together with FLAG-TR3 or EV along with pARE2-TATA-luc and treated with 20 µM or vehicle in the presence or absence of 10 nM DHT. (**G**) TR3 increases androgen-independent nuclear translocation of ARs. HEK 293T cells were transfected with GFP-AR-FL or GFP-AR-NTD together with TR3 or EV in the absence of androgen. Subcellular localizations of ARs and TR3 were detected as green fluorescent protein (GFP) and red Alexa Fluor568 signal, respectively. Nuclei were stained blue with TOPRO-3. Arrowheads indicate strong signals of nuclear-localized AR proteins in TR3-coexpressing cells. Images were acquired using a confocal microscope. Scale bars, 25 µm. (**H**) TR3 overexpression enhances coactivator recruitment to AR. PPC-1 cells were co-transfected with AR, SRC-2, and FLAG-TR3 or empty vector along with pARE2-TATA-luc and incubated with or without 1 nM DHT. Data are shown as mean ± SEM. *, *p* < 0.001; ns, not significant; one-way ANOVA with Tukey’s post-hoc test.

We then examined the effect of TR3 on the transcriptional activity of ARs (full-length AR, AR-FL; AR N-terminal domain, AR-NTD; and AR-V7) based on luciferase reporter assays. The AR-NTD lacking LBD was used to represent the ligand-independent nature of AR, and AR-V7 was used to mimic AR-V behavior in CWR22Rv1 cells. Overexpression of TR3 markedly enhanced both androgen-dependent and -independent transactivation of exogenous AR-FL, AR-NTD, and AR-V7 overexpressed in PPC-1 cells in a dose-dependent manner (Figure 5A–C and Figure S4A–C). In addition, overexpression of TR3 significantly enhanced the androgen-induced transactivation of endogenous AR in androgen-dependent LNCaP cells (Figure 5D). Blocking the function of TR3 using TR3-specific antagonist (DIM-C-pPhOH) impaired TR3-enhanced transactivation of AR-FL and AR-V7 (Figure 5E,F). DIM-C-pPhOH not only inhibited the function of TR3 but also reduced TR3 protein level (Figure 1G and Figure S1F).

The subcellular localization of ARs and recruitment of coactivators to AR target genes are important for AR transactivation. TR3 overexpression promoted the nuclear translocation of ARs even in the absence of androgen (Figure 5G). In addition, co-overexpression of TR3 facilitated the recruitment of AR coactivators, such as SRC-2, to the AR response element, synergistically enhancing the transactivation of AR (Figure 5H). Furthermore, the physical interaction between TR3 and androgen-independent AR-NTD (Figure S4D) suggests a molecular mechanism through which TR3 increases the activity of ARs in both an androgen-dependent and -independent manner. Collectively, these results show the profound effect of TR3 on the transactivation of ARs in prostate cancer, both in an androgen-dependent and -independent manner.

### 3.6. TR3 Overexpression Promotes the Proliferation and Mobility of Prostate Cancer Cells

**Figure 6:**
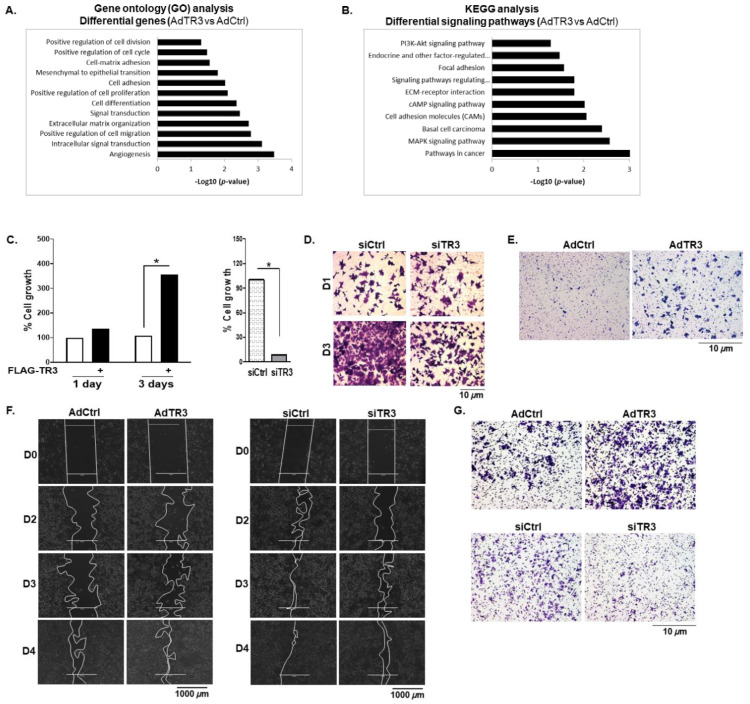
TR3 overexpression promotes the proliferation and mobility of androgen-independent prostate cancer cells. (**A**,**B**) TR3 overexpression enhances cellular signaling crucial for prostate cancer progression. RNA-Seq analysis was performed on CWR22Rv1 cells infected with AdTR3 or AdCtrl. The gene oncology (GO) (**A**) and KEGG (**B**) analysis of RNA-Seq data show upregulated expression of genes and enhanced signaling pathways. (**C**) TR3 regulates androgen-independent prostate cancer cell proliferation. Percent cell growth of TR3-overexpressing ((**C**), left) or TR3-knockdown ((**C**), right) CWR22Rv1 cells. Data are shown as mean ± SEM. *, *p* < 0.001; two-tailed *t*-test. (**D**) TR3 knockdown significantly reduced the viability of CWR22Rv1 cells. Cells were silenced using siTR3 or siCtrl for 1 day and 3 days, stained with crystal violet, and imaged using ZEISS microscopy at 20× magnification. (**E**–**G**) TR3 regulates androgen-independent prostate cancer cell mobility. Boyden Chamber migration assay of CWR22Rv1 cells infected with AdTR3 or AdCtrl (**E**). Cells were allowed to migrate for 24 h, stained with crystal violet, and imaged using ZEISS microscopy at 10× magnification. Scratch wound-closure assay of CWR22Rv1 cells infected with AdTR3 or AdCtrl ((**F**), left) and cells transfected with siTR3 or siCtrl ((**F**), right). Scratch-wounds were generated (day 0, D0) and the wound distances were monitored for 2 (D2), 3(D3), and 4 (D4) days, which were imaged using EVOS^®^ FL Cell Imaging System at 4× magnification. The boundary lines were simply drawn using shape tool from the PowerPoint. Invasion assays of CWR22Rv1 cells infected with AdTR3 or AdCtrl ((**G**), top) and cells transfected with siTR3 or siCtrl ((**G**), bottom). Cell invasion was allowed to occur for 48 h, stained with crystal violet, and imaged using ZEISS microscopy at 10× magnification.

RNA-Seq analysis showed the upregulation of genes that are involved in cell proliferation, migration, and invasion as well as PI3K/AKT and MAPK/ERK signaling pathways when TR3 is overexpressed (Figure 6A,B). The PI3K/AKT and MAPK/ERK signaling pathways are amplified as prostate cancer progresses into CRPC [2,27]. Therefore, we further explored the function of TR3 in cell proliferation and mobility, which are the downstream cell behaviors of AR signaling.

Overexpression of TR3 enhanced the proliferation of androgen-independent CWR22Rv1 cells (Figure 6C, left) as well as androgen-sensitive LNCaP cells under androgen-, outlaw IL6-, and forskolin-stimulated conditions (Figure S5A). In contrast, TR3 silencing significantly inhibited the proliferation of these prostate cancer cells (CWR22Rv1 and LNCaP) (Figure 6C, right; Figure S5B), which was confirmed using viability assays of TR3-knockdown CWR22Rv1 cells (Figure 6D).

To examine the effect of TR3 on cell mobility, we performed cell migration and invasion assays using CWR22Rv1 cells. TR3 overexpression markedly increased cell migration when examined by the Boyden chamber migration (Figure 6E) and scratch wound-closure (Figure 6F, left) assays. TR3 overexpression also enhanced CWR22Rv1 cell invasion (Figure 6G, top). Silence of TR3 resulted in a decrease in wound-closure based cell migration (Figure 6F, right) and cell invasion (Figure 6G, bottom). Collectively, these results suggest that TR3 increases the proliferation and mobility of prostate cancer cells.

### 3.7. TR3 Overexpression Enhances In Vivo Tumorigenesis of Androgen-Independent Prostate Cancer Cells

**Figure 7:**
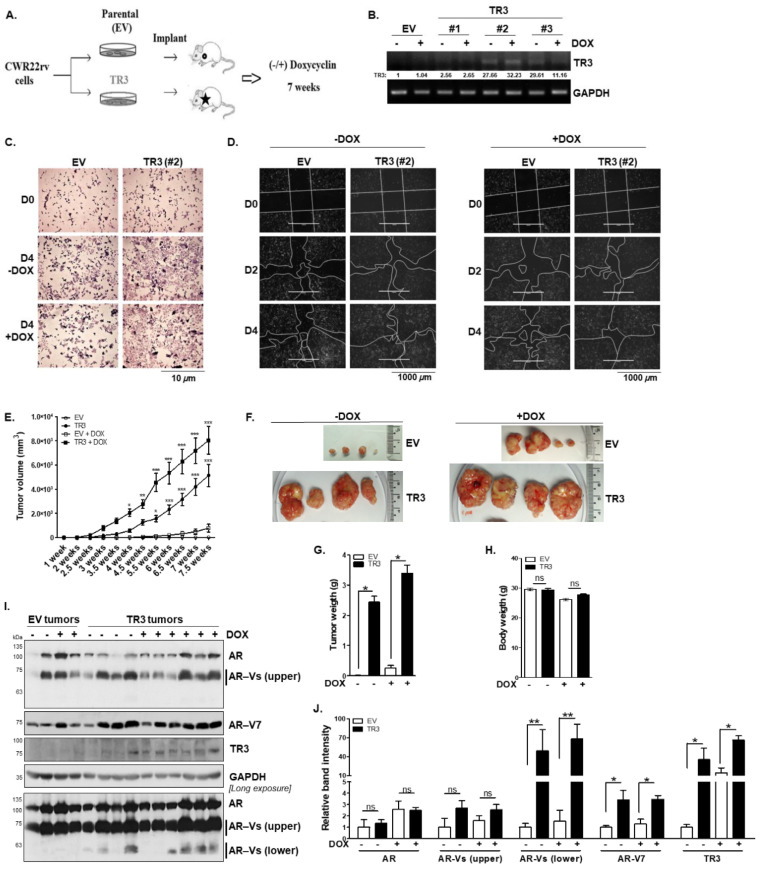
TR3 overexpression enhances in vivo tumorigenesis of androgen-independent prostate cancer cells. (**A**,**B**) Xenograft mouse model and establishment of a stable cell line overexpressing TR3. Schematic presentation of the experimental set-up for CWR22Rv1 xenograft mouse models (**A**). RT-PCR analysis showing TR3 expression in the inducible TR3-overexpressing CWR22Rv1 cell sublines (#1, #2, and #3) and the control (EV) treated with 2 µg/mL doxycycline (DOX) or vehicle (**B**). Values below gels indicate the relative band intensity of TR3 normalized to GAPDH. (**C**,**D**) A stable cell line overexpressing TR3 shows an increase in cell proliferation and mobility compared to the control (EV). Cell viability of stable EV and TR3 (#2) sublines (**C**). Cells were seeded for 12 h before treating DOX (D0), and maintained for 4 (D4) days in the absence or presence of 2 µg/mL DOX. Cells were then stained with crystal violet and imaged using ZEISS microscopy at 10X magnification. Scratch wound-closure assay of stable EV or TR3 (#2) sublines (**D**). Scratch-wounds were generated (day 0, D0) and the wound distances were monitored for 2 (D2) and 4 (D4) days in the absence ((**D**), left) or presence ((**D**), right) of DOX. (**E**–**J**) TR3 overexpression robustly promotes androgen-independent CWR22Rv1 xenograft tumor growth in vivo. The inducible TR3-overexpressing (TR3; subline #2) or control (EV) CWR22Rv1 cell clones were implanted into shoulders of 4-week-old male NOD.CB17-Prkdc^SCID^/J mice, and the mice were supplied with distilled water containing or not containing 2 µg/mL doxycycline (DOX) for 7 weeks. Tumor size and volume were measured three times per week for 7 weeks. Tumor volumes (**E**) measured for 7 weeks, and representative tumors (**F**) dissected from mice after 7 weeks. Data are shown as mean ± SEM, *n* = 2 independent biological in vivo experiments (*n* = 6 mice per each group). TR3 and TR3 + Dox tumor volumes were compared with EV and EV + DOX tumor volumes, respectively. *, *p* < 0.05; **, *p* < 0.01; ***, *p* < 0.001; two-way ANOVA with Bonferroni’s post-hoc test. Tumor weight (**G**) and body weight (**H**) measured after 7 weeks. Data are shown as mean ± SEM, *n* = 2 independent biological in vivo experiments (*n* = 6 mice per each group). *, *p* < 0.001; ns, not significant; two-tailed *t*-test. (**I**,**J**) Protein levels of AR and AR-Vs in xenograft tumors. Protein levels were analyzed via western blot analysis using anti-AR, anti-AR-V7, and anti-TR3 antibodies (**I**), and relative band intensity was measured using Image Studio Lite Ver. 5.2 (LI-COR, Inc.) (**J**). Data are shown as mean ± SEM. **, *p* < 0.01; *, *p* < 0.05; ns, not significant; two-tailed *t*-test. All uncropped western blot and gel original images are shown in Figure S7.

The effect of TR3 on prostate tumorigenesis was investigated using the inducible TR3-overexpressing (TR3) or control (EV) CWR22Rv1 xenograft mouse models (Figure 7A,B). The stable inducible TR3-overexpressing (TR3) CWR22Rv1 cells used for xenograft mouse models were evaluated for cell proliferation and migration. TR3 cells showed faster cell proliferation as well as cell migration than the control (EV), which were elucidated by cell viability (Figure 7C) and scratch wound-closure cell migration (Figure 7D) assays performed in the presence or absence of 2 µg/mL doxycycline (DOX).

With the xenograft mouse models, we observed that the average volume of TR3 tumors was approximately 10-fold larger than that of EV tumors after ~7-week treatment with DOX (Figure 7E,F). The average weight of tumors from TR3 mice was also approximately 10-fold greater than that of tumors from EV mice, although there were no significant differences in body weight (Figure 7G,H). We also observed a significant increase in tumor growth in TR3 tumors even without DOX treatment (Figure 7E–G). It could be due to leakage of the DOX inducible vector system, pUHDrtTA2S-M2, which has been improved to dox- or tet-ON system [28]. Otherwise, it might be due to the high basal expression of TR3 (Figure 7B) because TR3 expression was directed under the serum-inducible CMV promoter [29].

We further evaluated the protein expression levels of AR and AR-Vs as well as AR-V7 in xenograft tumors (Figure 7I,J). Unlike those of the full-length AR, the protein levels of short AR-Vs and AR-V7 were significantly increased in TR3 tumors compared to the control EV tumors (Figure 7I,J). These results suggest the pivotal role of TR3 in in vivo tumorigenesis of advanced CRPC cells.

## 4. Discussion

Constitutive transcriptional activity of AR-Vs is important for prostate cancer progression. However, the molecular mechanisms underlying the production of AR-Vs remain unclear. In the present study, for the first time, we found a novel function of TR3, which controls AR splicing events in prostate cancer cells. Overexpression of TR3 upregulated the expression of mRNAs and proteins of smaller AR-Vs. Consistent with this finding, TR3 overexpression altered the splicing junction rates of AR pre-mRNA, resulting in the enhancement of mRNA levels of AR-Vs, such as AR45, AR-V7, and AR-V1~4, in advanced CRPC cells (CWR22Rv1 and C4-2). We also demonstrated that TR3 overexpression alters the expression of certain splicing factors and that TR3 interacts with several known AR splicing factors (hnRNP A2B1 and hnRNP E1/E2) as well as the intron regions in AR pre-mRNA. Interestingly, TR3 functions in the RNA splicing process, while also regulating gene expression as a transcription factor. The association of TR3 with splicing factors and AR pre-mRNA might control the recruitment of spliceosomal complex components or identification of intron/exon boundaries.

Many splicing factors, including hnRNP A family (A0, A1/A1P10, and A2/B1) [8], hnRNP E1/E2 and HUR [7], proline-rich splicing factor PSF/SFPQ [30,31], and U2AF1/U2AF65 [4], are involved in the splicing of AR-V7. Among them, PSF regulates AR splicing events by interacting with spliceosomal complex components as an integrator [30,31]. It binds to the intronic region of AR transcripts and coordinates the complex formation of the spliceosome to promote the production of AR-V7 variant. Similar to PSF, TR3 directly or indirectly binds to the intronic region of AR transcripts at Reg4 and Reg8 and interacts with spliceosomal complex components, such as hnRNP A2B1 and hnRNP E1/E2. Many known RBPs contain the RNA recognition motif (RRM), KH, serine-rich, or poly-proline domains. Although the TR3 amino acid sequence does not contain RRM or KH domains, it has a putative poly-proline rich motif that shows high homology with that of PSF. These similarities suggest that TR3 may control AR splicing events as PSF does in prostate cancer cells.

The coupling of transcription and splicing processes is well-documented [20,21,22]. Transcription factors bound to the promoter or enhancers are likely involved in the recruitment of splicing factors to the site of transcription [20]. In addition, the C-terminal domain of RNA polymerase II (RNA pol II CTD) binds to some processing factors in the spliceosomal complexes and supports splicing factor recruitment to splice sites as shown with PSF and U2AF65-Prp19 complex [4,30,31,32]. Interestingly, TR3 also interacts with RNA pol II CTD [25]. Therefore, TR3 overexpression may lead to the formation of alternative spliceosomal complexes by recruiting splicing factors to unusual splice sites through its interaction with RNA pol II CTD in AR pre-mRNAs, thus promoting the production of AR-Vs. In addition, TR3 bound to the intronic regions of the AR gene may directly recruit some splicing factors, such as hnRNP A2B1 and hnRNP E1/E2, through protein–protein interactions. Otherwise, it may indirectly change the local chromatin structure, thus inhibiting the splicing between exon 3 and exon 4 of AR-FL, which eventually promotes the production of small transcripts, such as AR-V7 [33].

RNA-Seq analysis in the present study revealed that TR3 overexpression in CWR22Rv1 cells enhanced the expression of immune tolerance-related genes (Figure S6A) while decreasing the expression of immune defense-related genes, particularly inflammatory-related genes (Figure S6B,C). Additionally, IL23-RORɣ-STAT3 signaling axis was upregulated when TR3 was overexpressed (Figure S6D–**F**). It has been reported that IL23-RORɣ-STAT3 axis signaling is activated when the proportions of circulating mature myeloid-derived suppressor cells (PMN-MDSCs) are increased during CRPC progression. All together, TR3 may play a role in the alteration of immune responses to facilitate prostate cancer progression.

TR3 expression is upregulated in both primary prostate tumor and tissue adjacent to the tumor, and high expression level of TR3 in the tumor is associated with the short-term survival of patients. In addition, the overexpression of TR3 upregulated the expression of genes that are involved in cell proliferation, migration, invasion, angiogenesis, and pathways in cancer as well as PI3K/AKT and MAPK/ERK signaling pathways that are amplified as prostate cancer progresses into CRPC. Therefore, TR3 could be a therapeutic target to improve the outcome of prostate cancer treatment.

## 5. Conclusions

TR3 increases the androgen independence of AR signaling in prostate cancer cells by controlling AR expression, splicing events, and androgen-independent function and also stimulates tumorigenesis of prostate cancer cells. These results suggest that TR3 is a pivotal factor in the progression of prostate cancer to advanced castration-resistant form. Therefore, TR3 could be an alternative therapeutic target for the treatment of advanced prostate cancer.

## Data Availability

The data that support the findings of this study are contained within the article and available from the corresponding author upon reasonable request.

## Supplementary Materials

The following supporting information can be downloaded at: https://www.mdpi.com/article/10.3390/cancers14081911/s1, Figure S1: TR3 regulates AR and AR-V expression; Figure S2: TR3 overexpression alters the expression level of AR-Vs; Figure S3: TR3 overexpression alters the expression level of some factors involved in the splicing of AR pre-mRNA; Figure S4: TR3 overexpression enhances AR transactivation; Figure S5: TR3 affects the proliferation of prostate cancer cells; Figure S6: TR3 overexpression impairs the immune system and activates the IL23R/STAT3/RORC axis involved in prostate cancer progression; Figure S7: Uncropped western blot and gel original images; Table S1: Oligonucleotides; Table S2: Antibodies and sources, Supporting data: Uncropped western blot and gel images [34,35].

## Abbreviations

AR	androgen receptor
ARE	androgen-response element
AR-Vs	androgen receptor variants
AR-FL	full-length AR
AR-NTD	AR N-terminal domain
CE3	cryptic exon 3
CLIP	cross-linked RNA-immunoprecipitation
CRPC	castration-resistant prostate cancer
DBD	DNA-binding domain
ELAVL1/2	ELAV-like protein 1/2
hnRNP	heterogeneous nuclear ribonucleoprotein
IGV	integrative Genomic Viewer
LDB	ligand-binding domain
NTD	NH2-terminal transactivation domain
pre-mRNA	precursor mRNA
PMN-MDSCs	mature myeloid-derived suppressor cells
RBPs	RNA-binding proteins
RNA pol II CTD	C-terminal domain of RNA polymerase II
RRM	RNA recognition motif
ssRNA oligo	thirty-mer ssRNA labeled with biotin at the 5′ end
TRE	TR3 response element
