# Supplementary material for: TR3 Enhances AR Variant Production and Transactivation, Promoting Androgen Independence of Prostate Cancer Cells
Source: Cancers (Basel). 2022 Apr 10;14(8):1911. doi: 10.3390/cancers14081911 (PMC9031921; doi:10.3390/cancers14081911)
Supplement: Supplementary file 1 [file cancers-14-01911-s001.zip › cancers-1582727-supplementary.pdf]

cancers

MDPI

Supplementary Materials

TR3 Enhances AR Variant Production and Transactivation, Promoting Androgen Independence of Prostate Cancer Cells

Tuyen Thanh Tran and Keesook Lee

1. Supplementary Materials and Methods

1.1. Reagents

Forskolin (FSK; F3917), G418 disulfate salt (Geneticin; A1720), doxycycline (DOX; D9891) and chloroquine diphosphate salt (CQ; C6628) were purchased from Sigma-Aldrich (St Louis, MO, USA). The proteasome inhibitor carbobenzoxy-L-l eucyl-L-leucyl-L-leucinal (MG-132; M-1157) was purchased from A.G. Scientific, Inc. (San Diego, CA, USA). Recombinant human IL-6 (IL6; 206-IL-010) was purchased from R&D systems Inc. (Minneapolis, Minnesota, USA), 5α-dihydrotestosterone (DHT) was purchased from Sigma (Poole, UK). Enzalutamide (MDV-3100; SRP016825m) and Bicalutamide (BIC; SRP02002b) were purchased from Sequoia Research Products Ltd. (St James Close, Pangbourne, UK). 1,1-Bis(3′-indolyl)-1-(ρ-hydroxyphenyl)methane (DIM-C-pPhOH; 6377) was purchased from TOCRIS Bioscience (Bristol, UK).

1.2. Plasmids

pARE2-TATA-Luc, GAL4.AR-LBD658-919, VP16.AR1-660, 5xGAL4-luc3, pcR3.1 SRC-1, pcDNA3.AR, pcDNA3.AR-NTD, pEGFP-AR, and pEGFP-AR-NTD have been previously described [34]. pcDNA3.AR-V7 was constructed to mimic AR-Vs in CWR22Rv1 cells. Exons 2–8 were excised from pcDNA3.AR using *Kpn*I and *Xba*I and then exons 2 and 3 and CE3 amplified from CWR22Rv1 cDNA library were inserted. pcDNA3.FLAG-TR3 was constructed by inserting TR3 PCR fragment into the pcDNA3.FLAG expression vector within *Eco*RI and *Eco*RV sites. The primers for PCR to construct the expression vectors are listed in Table S1.

1.3. Cell Transfection and Reporter Assays

Cells were transiently transfected with expression constructs or siRNAs, and a luciferase reporter construct together with pCMV-LacZ or pRSV-LacZ (Clontech) using lipofectamine™ 2000 reagent (11668-019; Invitrogen, Carlsbad, CA) with minor modifications of manufacturer's instruction. Transfected cells were starved in media containing 5% cFBS for 24 h before stimulating with androgen, growth factors, or antagonists. Cells were then lysed in luciferase lysis buffer [0.2M Tris-Cl (pH 8.0), 0.2% Triton X100, and 1% NP-40] at RT for 15min. Luciferase activity was then analyzed in Beetle Luciferin (E1603; Promega Co., Madison, Wisconsin, USA) using a Centro XS3 LB960 Luminometer (Berthold Technologies GmbH & Co. KG, 75323 Bad Wildbad, Germany) and normalized to β-galactosidase activity read by Versa Max microplate reader (Molecular Devices, LLC., San Jose, CA). The duplet siRNA sequences for silencing study are listed in Table S1.

1.4. RNA Isolation, RT-PCR and qPCR

Total RNA was isolated from prostate cancer cell samples by using TRI reagent® [TR 118; Molecular Research Center (MRC), Inc. Cincinnati, OH]. Reverse transcription was performed using Oligo d(T)$_{15}$ (EBT-1523; ELPiS, Taejeon, Korea) and M-MLV Reverse transcriptase kit (M1705; Promega, Madison, Wisconsin, USA). The mRNA levels were analyzed by RT-PCR using Taq polymerase and quantified by qPCR using TOPreal™

qPCR 2X preMIX (RT501M; Enzynomics, Daejeon, Republic of Korea). The primer sequences for analysis of gene expression profile and alternative transcripts of ARs are listed in Table S1.

1.5. Western Blot Analysis

Western blot assays were performed as previous described [34]. Proteins were separated by SDS-PAGE, and then transferred onto a nitrocellulose blotting membrane (10600004; Amersham™ Portran™ Preminum 0.2 μM; GE healthcare, Little Chalfont, UK). The membrane was blocked, incubated with primary antibodies at 4°C overnight, and then incubated with secondary antibodies for 1 h. Band signals were visualized on X-ray films (28906839; Amersham HyperfilmTM ECL; GE Healthcare, Little Chalfont, UK) or scanned by iBright 1500 imaging system (ThermoFisher Scientific) with ECL™ western blotting analysis system (RPN2109; GE Healthcare, Little Chalfont, UK). The antibodies are listed in Table S2.

1.6. CoImmunoprecipitation (Co-IP)

Co-IP assays were performed as previous described [34]. Cells were sonicated in 25 mM Tris-Cl (pH 8.0) buffer containing protease inhibitors. Supernatant was collected and then processed to Co-IP assays using protein A/G PLUS-Agarose beads (Santa Cruz Biotechnology, Dallas, Texas, USA) and anti-hnRNP A2B1, anti-hnRNP E1/E2, anti-HUR, anti-AR or anti-TR3 antibodies. Proteins were then detected by western blot analysis.

1.7. Chromatin Immunoprecipitation (ChIP)

Chromatin immunoprecipitation assay was performed as previously described [34]. ChIP assays were performed using CWR22Rv1 cells infected with AdTR3 or Ad-Ctrl. Recruitment of TR3 protein to putative TR3 binding sites within the promoter and intron regions of AR gene (P and A–C regions) was determined by ChIP assays using anti-TR3 antibody. Changes in TR3 enrichment at putative TR3 binding sites (P, A, B, and C) were examined using PCR. The loading control (Ctrl) was *β*-actin. The primers for ChIP assay are listed in Table S1.

1.8. Generation of TR3-Overexpressing Adenoviral (AdTR3) Constructs

TR3-overexpressing adenovirus (AdTR3) was generated as previously described [35]. FLAG-TR3 fragment was excised from pcDNA3.FLAG-TR3 using *Kpn*I and *Xho*I and inserted into pAdTrack.CMV within *Kpn*I and *Xho*I sites to generate pAdTrack.CMV.FLAG-TR3. To generate the stable homologous recombinant adenovirus, purified *Pme*I-linearized pAdTrack.CMV.FLAG-TR3 was introduced into BJ5183 bacterial cells harboring the supercoiled backbone vector (AdEasy-1 cells). The recombinant adenoviral construct was screened using *Pac*I digestion and then transiently transfected into recombinant adenovirus E1-expressing HEK-293 (AD-293) cells (Agilent Technologies, Inc., Santa Clara, CA 95051, USA) to generate the first virus generation (AdTR3). Viruses were further amplified and purified for infection experiments.

1.9. Subcellular Localization

HEK 293T cells were transiently transfected with GFP-AR-FL or GFP-AR-NTD, with or without TR3, for 12 h. The cells were fixed using 3.7% paraformaldehyde in PBS, blocked with BSA, incubated with primary mouse anti-TR3 antibodies overnight at 4 °C, and then incubated with secondary goat anti-mouse Alexa Fluor568. Nuclei were stained using TOPRO-3. Subcellular localizations of ARs (green GFP signal) and TR3 (red Alexa Fluor568 signal) were analyzed using a Nikon A1 laser-scanning Leica TCS SPE confocal microscope equipped with an ACS APO ×63/1.30 numerical aperture immersion objective. Images were analyzed using the ImageJ software (http://imagej.nih.gov/ij, ImageJ 1.46r, Wayne Rasband, National Institutes of Health, USA).

1.10. Protein Purification

HEK 293T cells were infected with AdTR3 for 24 h and harvested for the purification of TR3 and splicing factors (hnRNP A2B1, hnRNP E1/E2, and HUB/HUR). Cell pellets were sonicated in 50 mM Tris-Cl (pH 7.5) containing protein inhibitors and the supernatant was collected. Proteins were purified through two-step gradient anion-cation exchange chromatography (Q-Sepharose and SP-Sepharose). The Q-sepharose was equilibrated with equilibrium buffer [50 mM Tris-Cl (pH 8.5) containing protein inhibitors], while SP-sepharose was equilibrated with equilibrium buffer [50 mM Tris-Cl (pH 6.0) containing protein inhibitors]. Unbound proteins were washed by washing buffer (equilibrium buffer containing 50mM NaCl). Bound proteins were eluted by gradient of buffer A (50 mM Tris-Cl containing protein inhibitors) and buffer B (50 mM Tris-Cl containing 1M NaCl and protein inhibitors). The purity of all unbound and bound fractions were analyzed by Coomassie Brilliant Blue R-250 stained SDS-PAGE. The fractions containing TR3 or each splicing factor (hnRNP A2B1, hnRNP E1/E2, and HUB/HUR) were confirmed through western blot analysis, stored in the presence of 0.5% sucrose for protein stabilization, and then used in in vitro RNA-protein interaction assays.

1.11. Cell Proliferation and Mobility Assays

For cell proliferation assays, cells were transfected with expression constructs or siRNA, or infected with adenovirus. After 12 h starvation, cells were treated with 1 nM DHT, 100 ng/ml IL6, or 50 μM FSK for 24-72 h. The medium was replaced each day using fresh media containing inducers. The percent cell growth and cell numbers were obtained using MTS assays, which were measured using a microplate reader system at λ of 490 nm.

For cell viability assays, CWR22Rv1 cells were infected with AdTR3 or AdCtrl, or transfected with siTR3 or siCtrl. Cells were maintained for 24 h, seeded on coverslips (10^4 cells per well) for 24–72 h, and then processed for cell viability assays. Cell viability assays were also performed with the stable CWR22Rv1 (EV and TR3) sublines, which were cultured for 4 days in the absence or presence of 2 μg/ml DOX. Three random fields of 0.5% crystal violet-stained cells were imaged using ZEISS microscopy at 10X or 20X magnification.

For scratch wound-closure assays, CWR22Rv1 cells were infected with AdTR3 or AdCtrl, or transfected with siTR3 or siCtrl. Cells were maintained for 24 h and then seeded in 12-well plates (16 x 10^4 cells per well). After 12 h seeding, scratch wounds were generated (day 0, D0). The floating cells were removed through replacement with fresh medium. The scratch spaces were monitored and imaged daily using EVOS® FL Cell Imaging System (Thermo Fisher Scientific, MA 02451, USA) at 4X magnification. Scratch wound-closure assays were also performed with the stable CWR22Rv1 (EV and TR3) sublines.

For cell invasion and Boyden Chamber migration assays, TR3-overexpressing or -silenced CWR22Rv1 cells were seeded onto a 24-well Costar chamber comprising an 8-μm polycarbonate membrane, which was precoated with 20% phenol red-free Matrix gel (8×10^4 cells per well) for invasion assay or without for Boyden Chamber migration assay. Cells were allowed to grow and invade for 48 h or migrate for 24 h and then stained using 0.5% crystal violet. Cells on the inner side of the chamber were gently removed by scraping with a cotton swab and rinsed several times with PBS to remove excess dye. Three random fields of stained cells were imaged using ZEISS microscopy at 10X magnification.

1.12. Data Processing

The Gene Expression Omnibus (GEO) profile of TR3 is available in NCBI's GEO profile https://www.ncbi.nlm.nih.gov/sites/GDSbrowser?acc=GDS2545, the public microarray dataset accession [GDS2545] and is accessible through https://www.ncbi.nlm.nih.gov/geo/query/acc.cgi?acc=GSE6919, GEO Series accession number [GSE6919]. The relative gene expression in fold change is shown as mean ± SEM. *****, $p<0.001$; ns, not significant; one-way ANOVA analysis with Tukey's post hoc test.

The gene expression profiles of TR3 (NSG00000123358-NR4A1) and AR (ENSG00000169083-AR) along with Time after diagnosis (Years) in prostate cancer tissues

(*n*=494) were obtained The Cancer Genome Atlas (TCGA) (https://tcga-data.nci.nih.gov/tcga/). RNA-seq data were reported as median Fragments Per Kilobase of exon per Million reads (FPKM). Correlation Pearson r or Spearman r and *p* values were analyzed using correlation analysis with two-tailed Pearson or two-tailed Spearman test, respectively.

2. Supplementary Figures and Supplementary Figure Legends

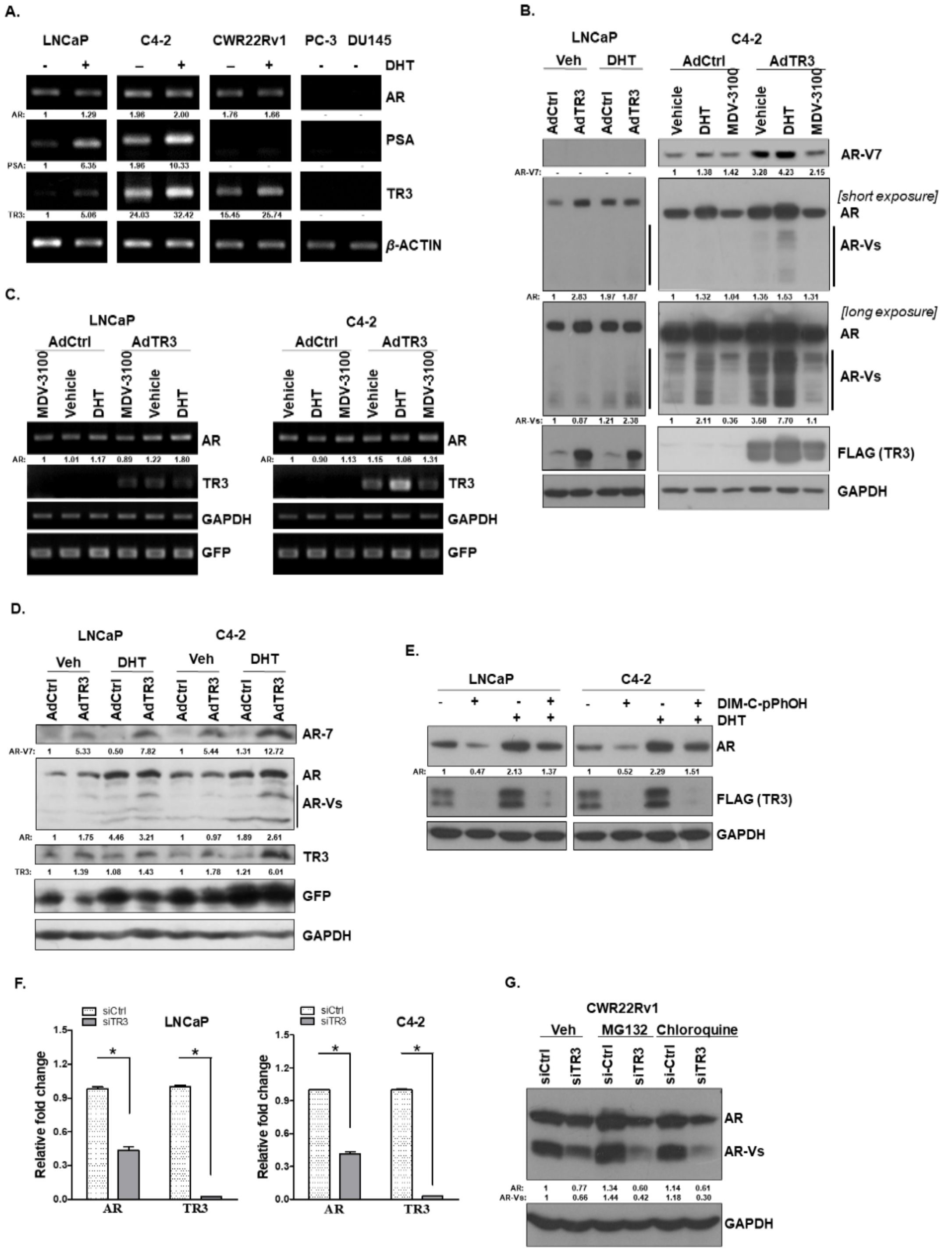

Figure S1. TR3 regulates AR and AR-V expression. **(A)** RT-PCR analysis showing mRNA levels of TR3, AR, and AR-target gene PSA in prostate cancer cell lines treated with 10 nM DHT or vehicle. *β*-ACTIN was used as a loading control. (**B-D**) TR3 overexpression increases the expression levels of AR and AR-Vs. Western blot (**B**) and RT-PCR (**C**) analysis showing the protein and mRNA levels of AR, AR-V7, and TR3 in LNCaP and C4-2 cells, which were infected with AdTR3 or AdCtrl and

treated with DHT, MDV-3100 or vehicle for 24 h. (**D**) Representative western blot analysis showing the protein levels of AR, AR-V7, and TR3 in LNCaP and C4-2 cells infected with AdTR3 or AdCtrl in the absence or presence DHT for 3 days. (**E)** Representative western blot analysis showing AR and TR3 protein levels in LNCaP and C4-2 cells, which were overexpressed with TR3 and treated with 20 μM of TR3-specific antagonist (DIM-C-pPhOH) in the presence or absence of 10 nM DHT. (**F)** TR3 silencing causes a decrease in AR mRNA levels. qPCR analysis showing AR and TR3 mRNA levels in LNCaP and C4-2 cells transfected with siTR3 or siCtrl. Data are shown as mean ± SEM. *, p<0.001; one-way ANOVA with Tukey's post hoc test. (**G**) The decreased AR and AR-V protein levels in TR3-silenced CWR22Rv1 cells were not restored following treatment with MG-132 or chloroquine for 8 h. GAPDH was used as a loading control. Values below gels and blots indicate the relative band intensity of each gene normalized to actin or GAPDH.

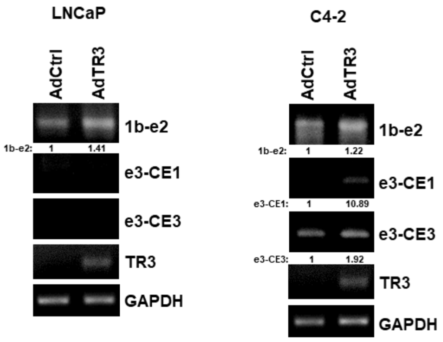

Figure S2. TR3 overexpression alters the expression level of AR-Vs. RT-PCR analysis showing the levels of mRNA containing alternative exons found in AR-Vs in LNCaP and C4-2 cells infected with AdTR3 or AdCtrl. Values below gels indicate the relative band intensity of each exon junction normalized to GAPDH.

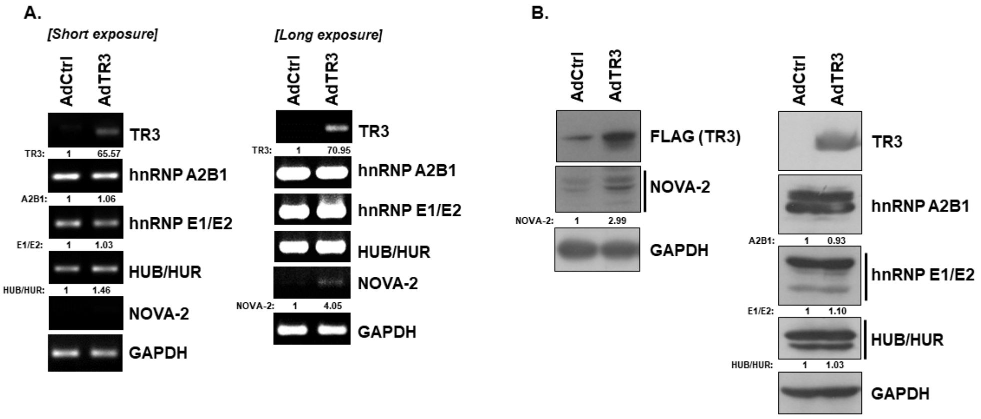

Figure S3. TR3 overexpression alters the expression level of some factors involved in the splicing of AR pre-mRNA. (**A**) RT-PCR analysis presenting mRNA levels of several splicing factors (hnRNP A2B1, hnRNP E1/E2, HUB/HUR, and NOVA-2) in CWR22Rv1 cells infected with AdTR3 or AdCtrl. (**B)** Representative western blot analysis showing the protein levels of several splicing factors (NOVA-2, hnRNP A2B1, hnRNP E1/E2, and HUB/HUR) in CWR22Rv1 cells infected with AdTR3 or AdCtrl. Proteins were detected by western blot analysis using anti-TR3, anti-FLAG, anti-NOVA-2, anti-hnRNP A2B1, anti-hnRNP E1/E2, and anti-HUR antibodies. Values below gels and blots indicate the relative band intensity of each gene normalized to GAPDH.

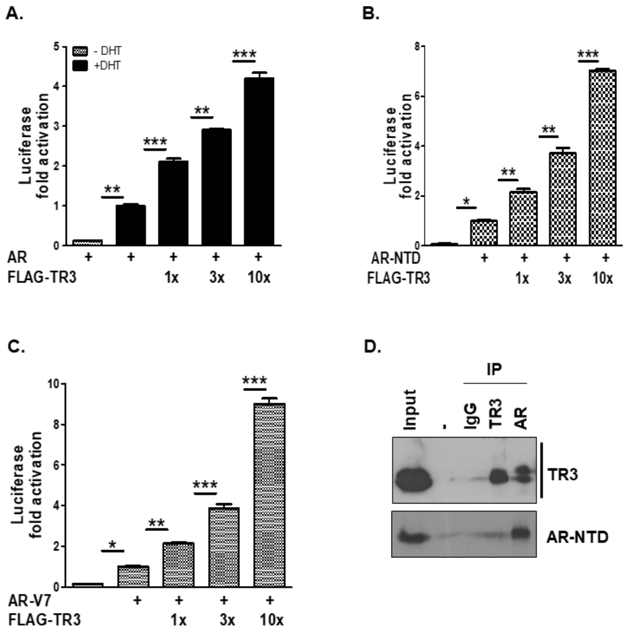

Figure S4. TR3 overexpression enhances AR transactivation. (**A-C)** TR3 overexpression enhances the transactivation of ARs in a dose-dependent manner. PPC-1 cells were transfected with full-length AR (AR-FL) (**A**), AR *n*-terminal domain (AR-NTD) (**B**), or AR-V7 (**C**) together with different amounts of FLAG-TR3 expression construct and pARE2-TATA-luc and incubated with or without 1 nM DHT. Luciferase activity was normalized to that of *β*-galactosidase. Data are shown as mean ± SEM. *, $p<0.05$; **, $p<0.01$; ***, $p<0.001$; one-way ANOVA with Tukey's post hoc test. (**D)** TR3 physically interacts with AR-NTD. PPC-1 cells were co-transfected with AR-NTD and TR3 expression constructs. Physical interaction between TR3 and AR-NTD was examined through coimmunoprecipitation performed using anti-TR3 or anti-AR antibodies. Proteins were detected using western blot analysis using anti-TR3 and anti-AR antibodies.

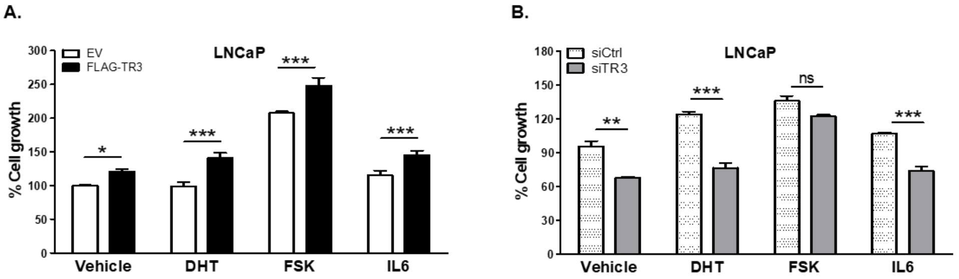

Figure S5. TR3 affects the proliferation of prostate cancer cells. TR3 expression was overexpressed (**A**) or silenced (**B**) in LNCaP cells. Cells were treated with 1 nM DHT, 50 ng/ml IL-6, 50 *μ*M FSK, or vehicle. Cell growth was assessed using MTS assays. Data are shown as mean ± SEM. *, $p<0.05$; **, $p<0.01$; ***, $p<0.001$; ns, not significant; two-tailed t-test.

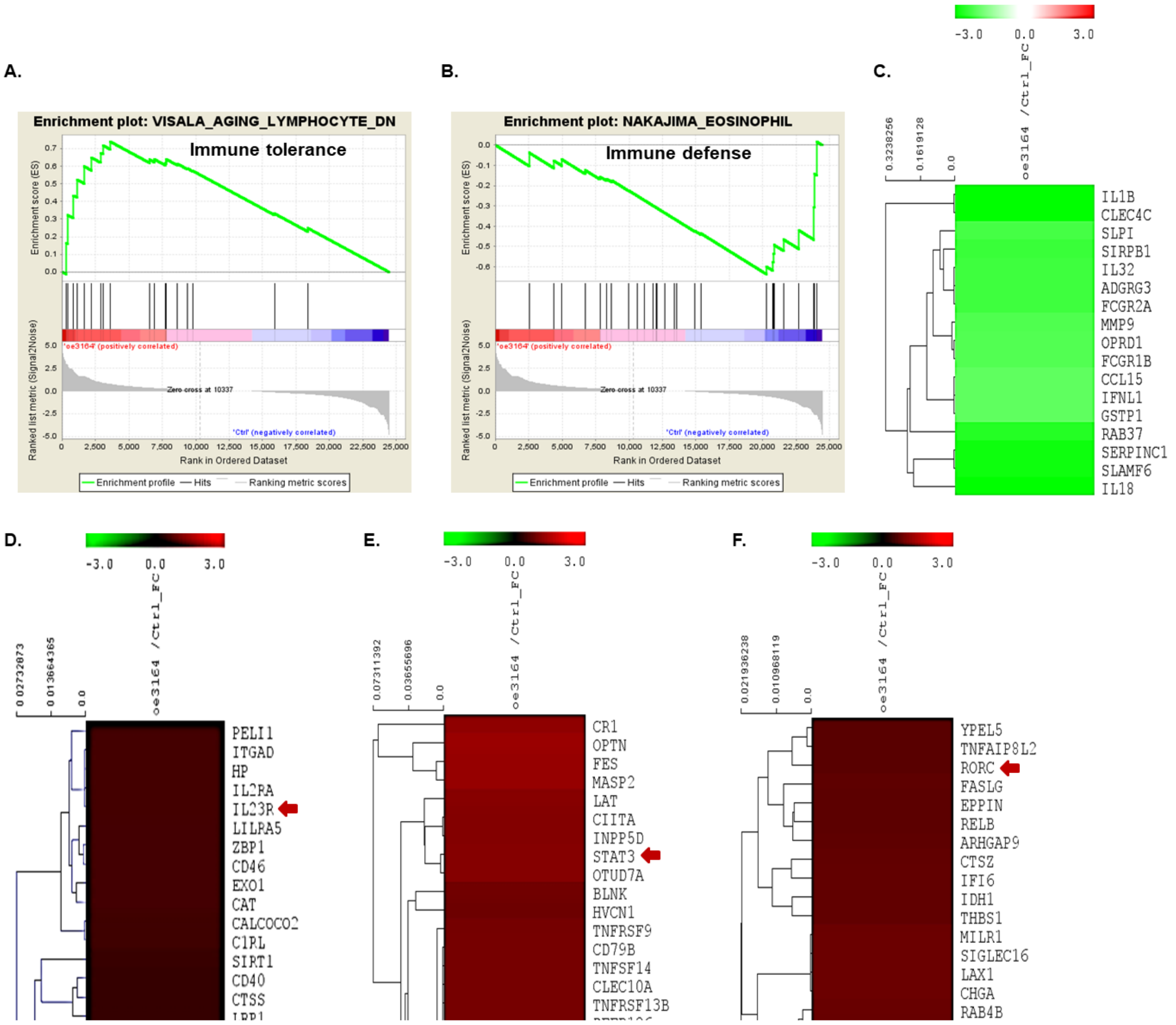

Figure S6. TR3 overexpression impairs the immune system and activates the IL23R/STAT3/RORC axis involved in prostate cancer progression. (**A-B)** Gene enrichment analysis (DAVID) of RNA-Seq data showing the upregulated expression of immune tolerance genes (**A**) and the downregulated expression of inflammatory genes (**B**) when TR3 was overexpressed in CWR22Rv1 cells. (**C-F)** Heatmaps (MeV) presenting the downregulated expression of inflammatory genes (**C**) and the up-regulated expression of IL23R (**D**), STAT3 (**E**), and RORC (**F**) in TR3-overexpressing CWR22Rv1 cells compared with the control.

Figure 1C (blot)

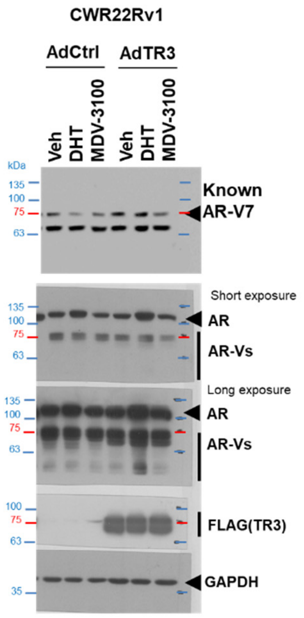

Figure 1 D (gel)

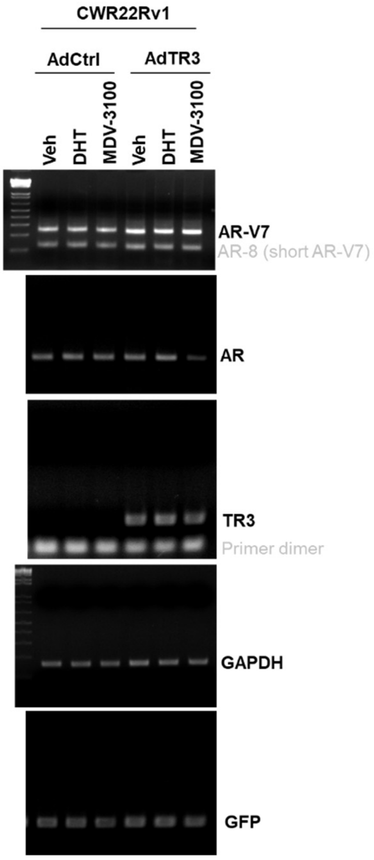

Figure 1F (blots)

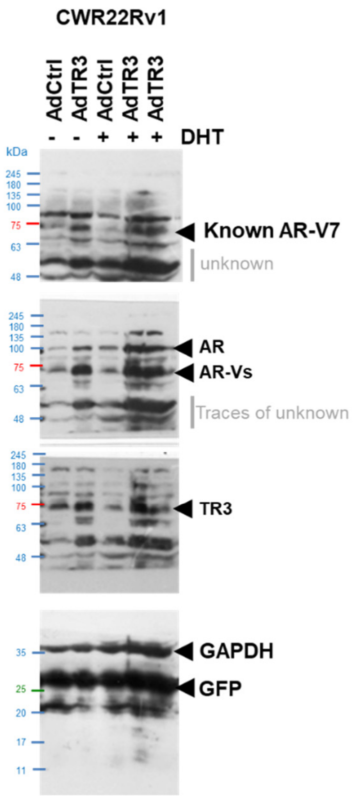

Figure 1G (blot)

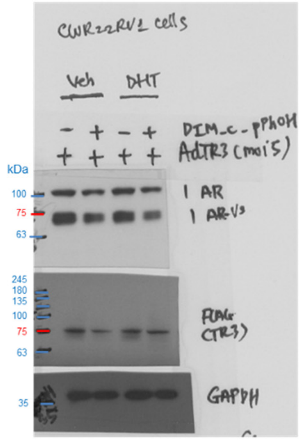

Figure 1H (blot)

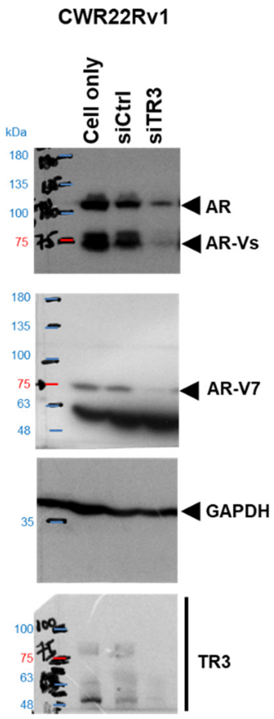

Figure 2B (gel)

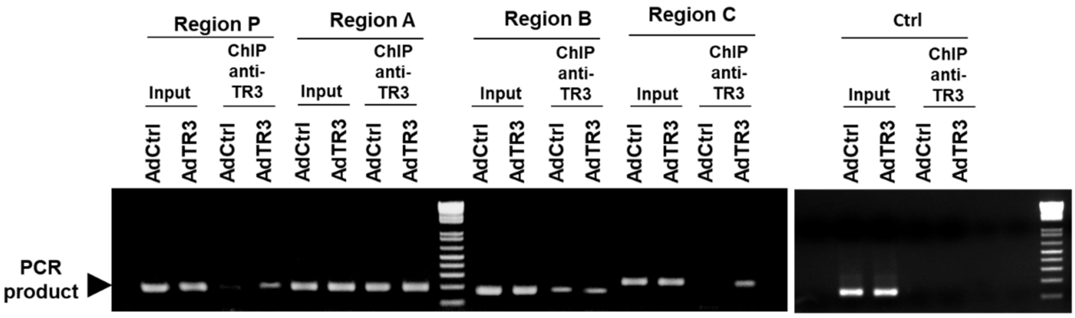

Figure 3C (blot)

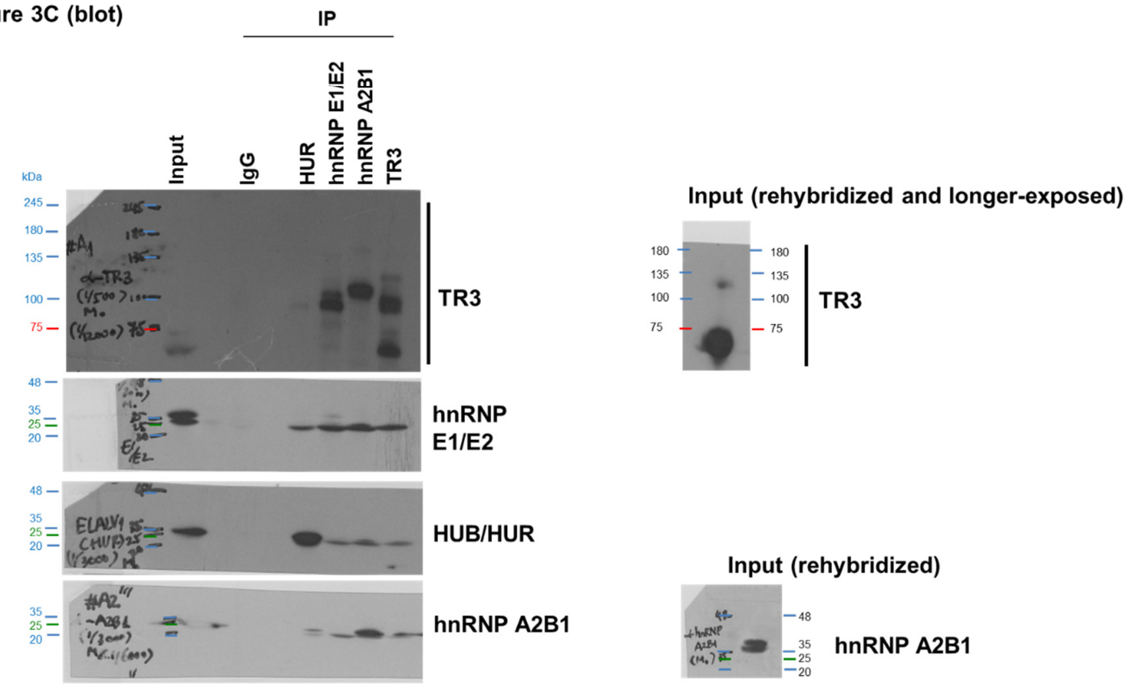

Figure 4B (gel)

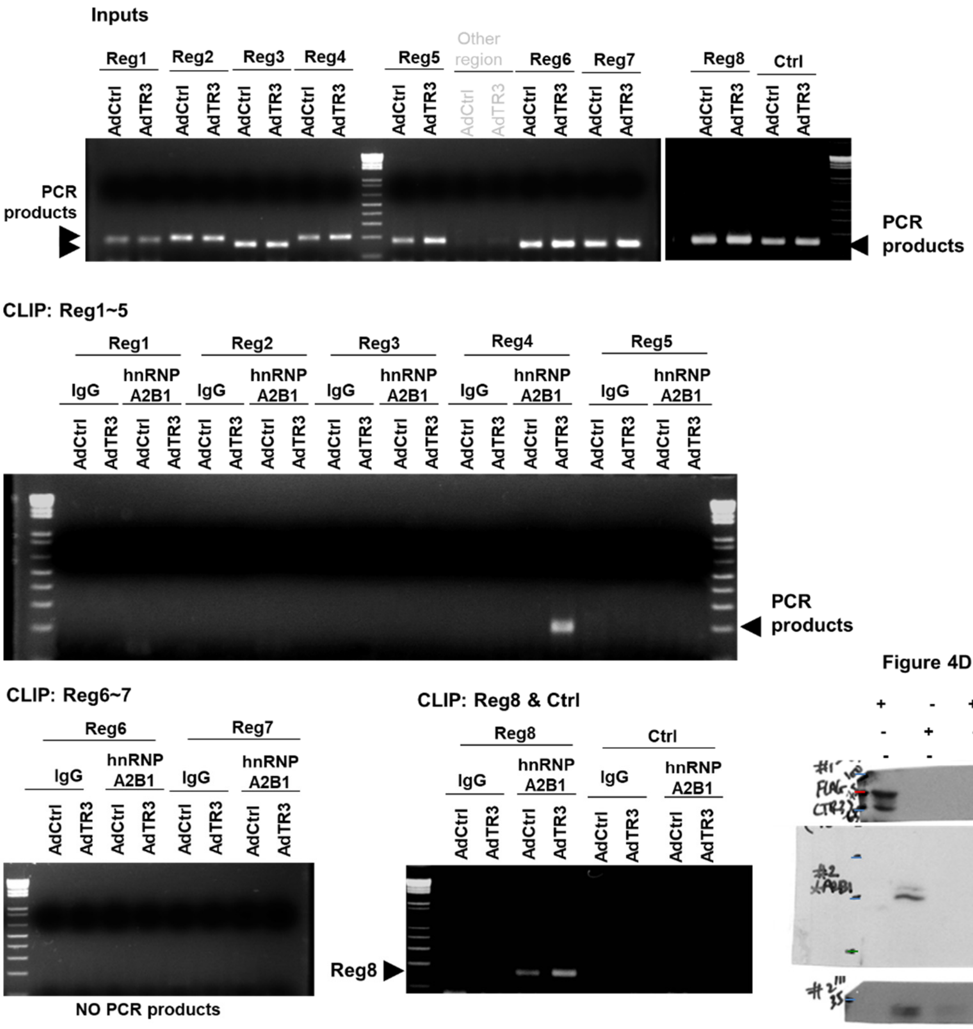

Figure 4D (blot)

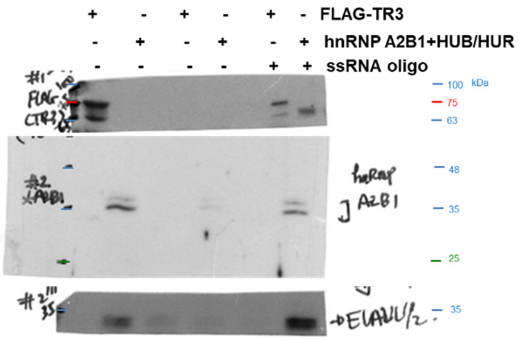

Figure 7I (blot)

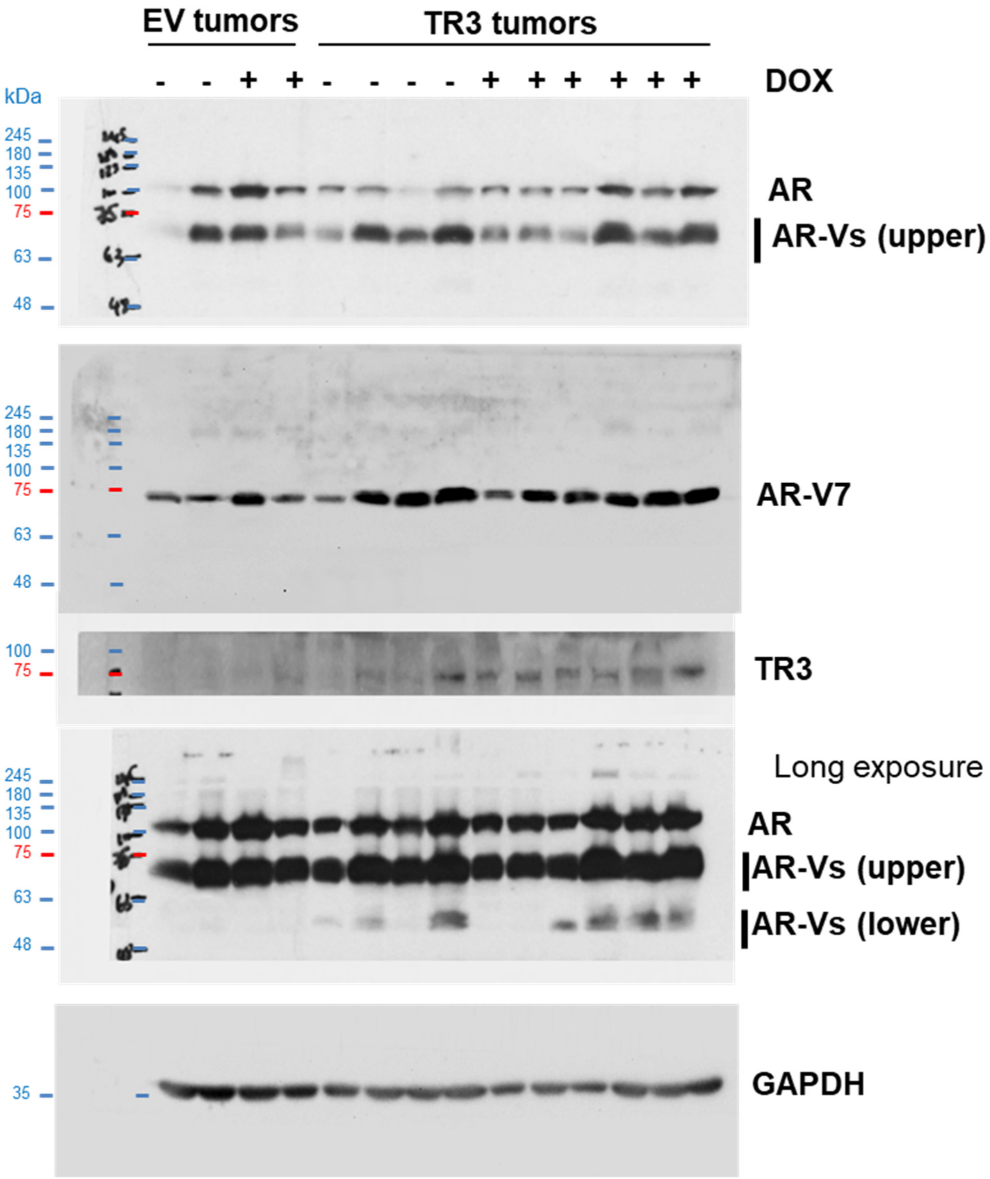

Figure S1A (gel)

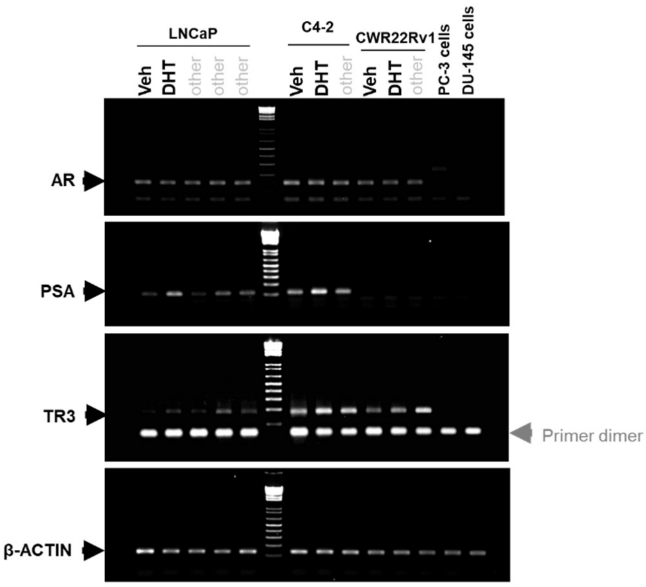

Figure S1B (blot)

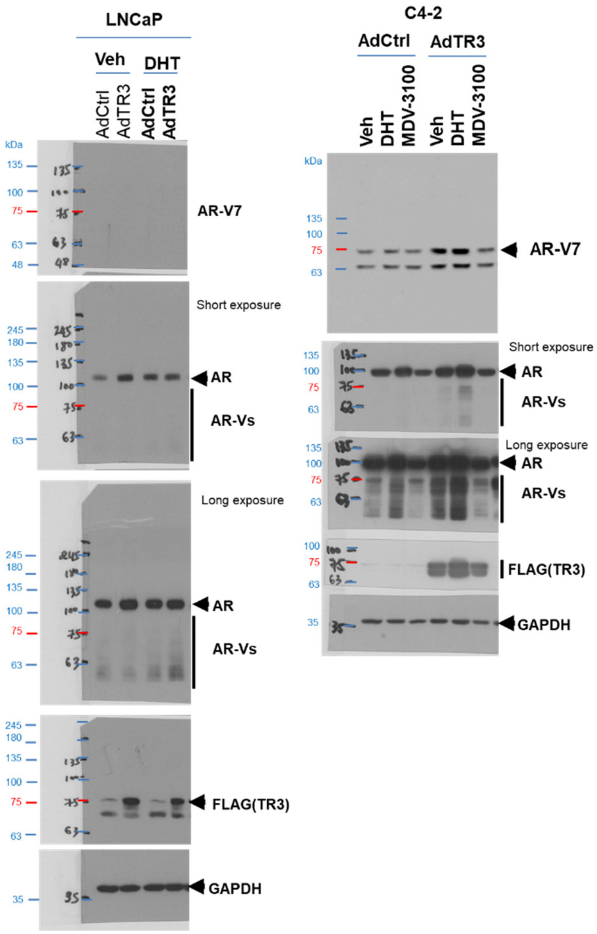

Figure S1C (gel)

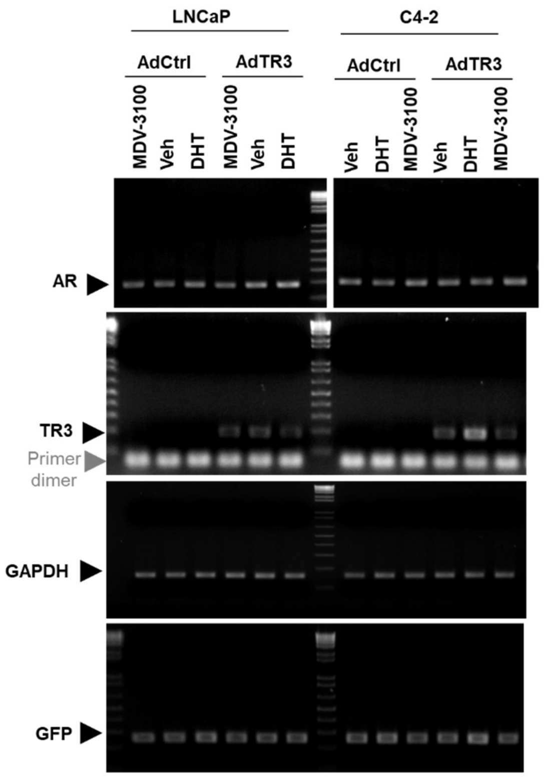

Figure S1D (blot)

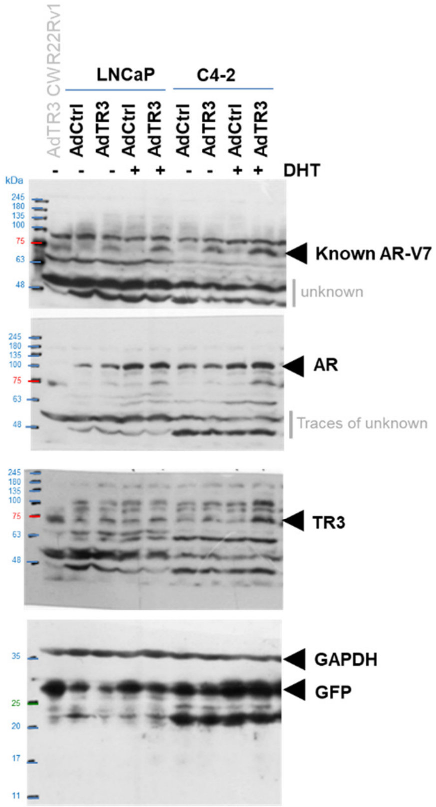

Figure S1E (blot)

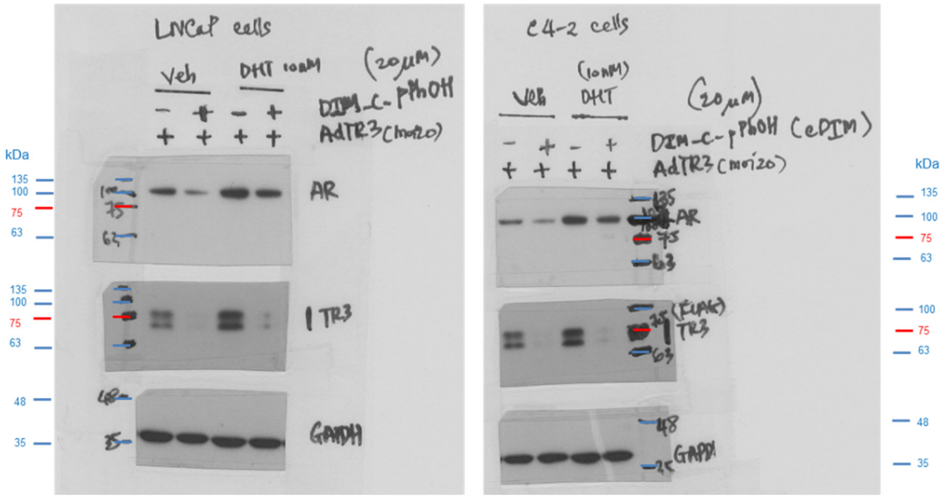

Figure S1G (blot)

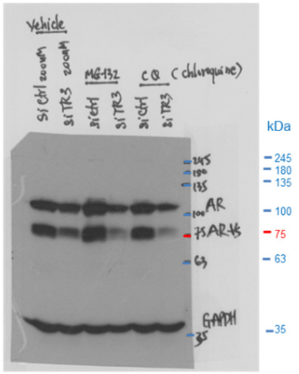

Figure S2 (gel)

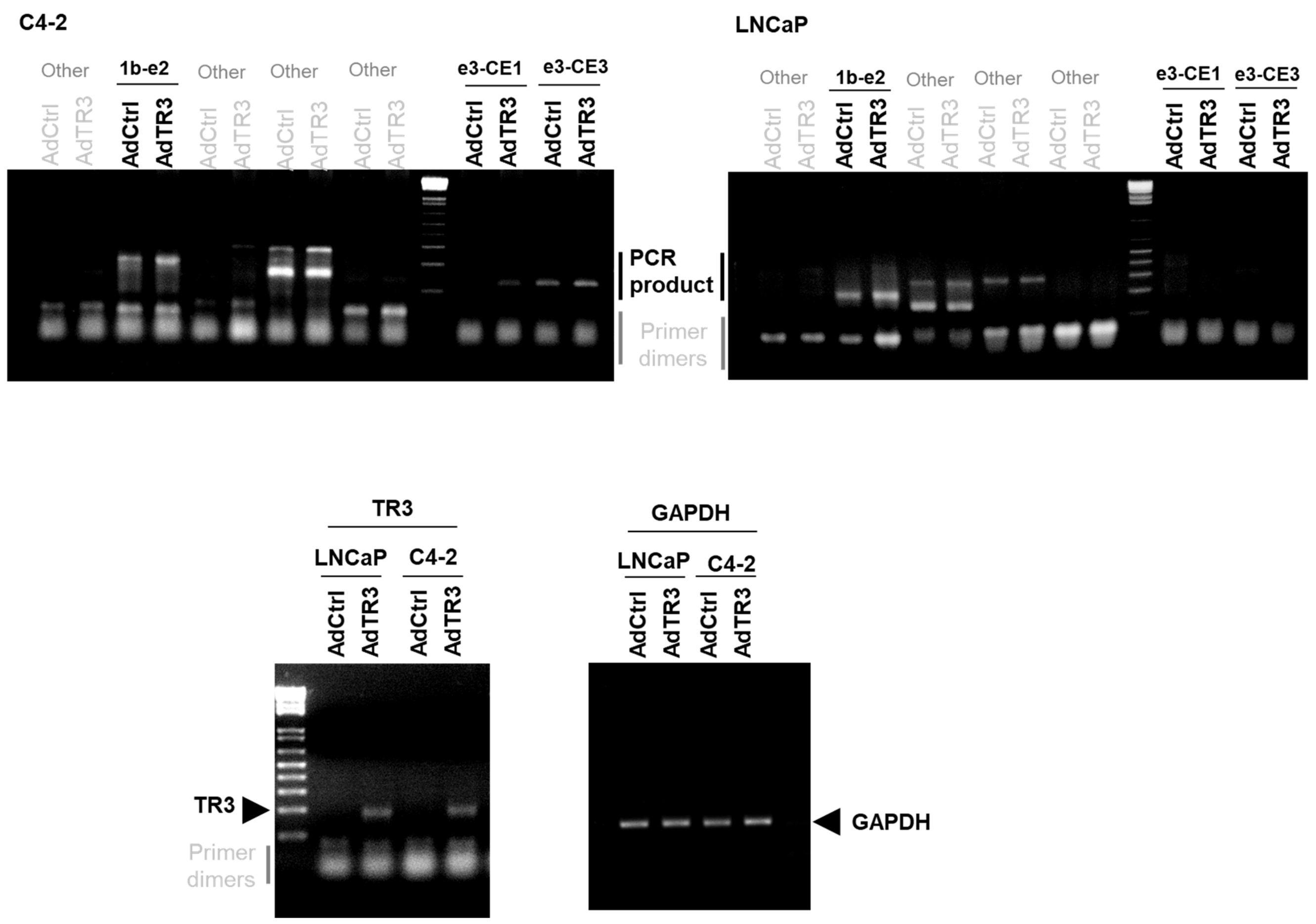

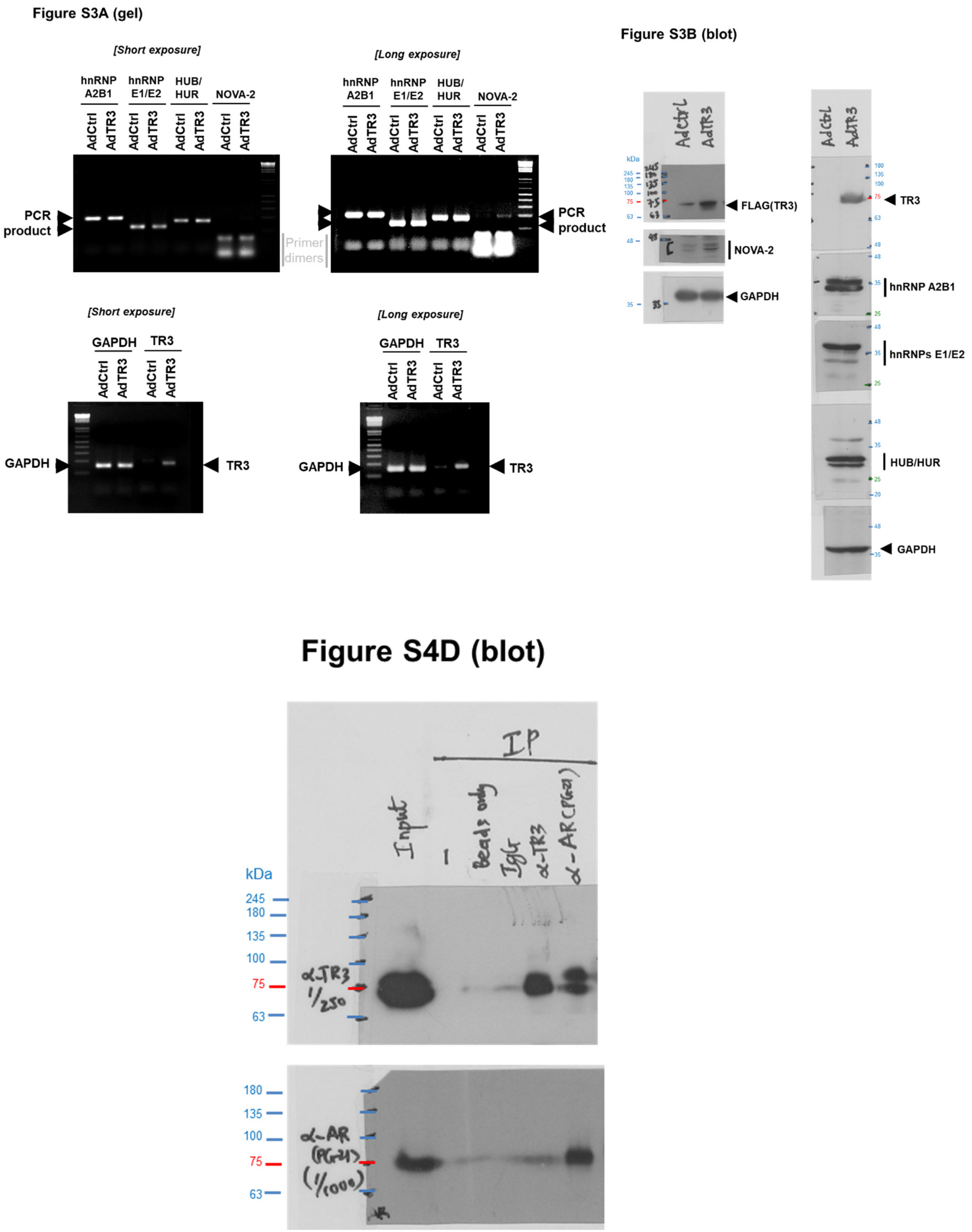

Figure S7. Uncropped western blot and gel original images.

3. Supplementary tables

Table S1. Oligonucleotides.

Cloning	AR-V7-Forward: ATAGGTACCCTGGCGGCATGGTGAG<br>AR-V7-Reverse: ATATCTAGATCAGGGTCTGGTCATTTTGAGATGCTTGCA<br>AdTR3-Forward: CGTGAATTCCCCTGTATCCAAGCCCAATAT<br>AdTR3-Reverse: ATAGATATCTCAGAAGGGCAGCGTGTCCAT
ChIP	P Forward: CAGAGATGAAATAAATGGGCAGATG<br>P Reverse: TCCTCTGCAACTTCAAGAGGA<br>A Forward: GACATGATGAATGTGAACATCCTTGA<br>A Reverse: CCAAGTTACTTAGGGTAAAAGCCATC<br>B Forward: ACACTTTCGAAAACATGGGTATAGAC<br>B Reverse: GATGGTCTGCTTTTGATCATTAATGC<br>C Forward: GATCGAATCAGCTACTGAAGCTTG<br>C Reverse: CCATCGCAAAGAAGTTAAAAACCTTG<br>Ctrl Forward: TCCTCCTCTTCCTCAATCTCG<br>Ctrl Reverse: AAGGCAACTTTCGGAACGG
CLIP	Reg1 Forward: AAAGAAAGTGGTCTCTGGGTGCTGA<br>Reg1 Reverse: GTGAGTGCAAATCCTGTGAAGTCTTTAC<br>Reg2 Foward: CAACCCACTGTGTATTGCAGAATGTTTTA<br>Reg2 Reverse: TTTCCCTGGTTCCCTGTACAAAGTC<br>Reg3 Foward: AGAGATGGGCATATTCCTTGTTTGAATG<br>Reg3 Reverse: CTCCCCTTACTGCCTTCCTTATATG<br>Reg4 Foward: CTGCAGTTGGAATTTACATTTCCAAAGC<br>Reg4 Reverse: CAACCCATTTTGACTAGAGACCTGAG<br>Reg5 Foward: ATTGTGGTATCTGTATGTGGACCCTG<br>Reg5 Reverse: GATACTGCAGTTTTGAACACTGCAAATC<br>Reg6 Forward: GACATGATGAATGTGAACATCCTTGA<br>Reg6 Reverse: CCAAGTTACTTAGGGTAAAAGCCATC<br>Reg7 Forward: ACACTTTCGAAAACATGGGTATAGAC<br>Reg7 Reverse: GATGGTCTGCTTTTGATCATTAATGC<br>Reg8 Forward: GATCGAATCAGCTACTGAAGCTTG<br>Reg8 Reverse: CCATCGCAAAGAAGTTAAAAACCTT

Table S1. Oligonucleotides (*Continue*).

RT-PCR and qPCR	e1-AR Forward: GAAATGGGCCCCTGGATG<br>e2-AR Reverse: CATCTCCACAGATCAGGCAGG<br>e3-AR Forward: TGCACTATTGATAAATTCCGAAGG<br>CE1-AR Reverse: CAAACACCCTCAAGATTCTTTCAG<br>CE3-AR Reverse: GTCATTTTGAGATGCTTGCAATTG<br>e4-AR Reverse: TTCTGGGTTGTCTCCTCAGT<br>1b-AR Forward: ATGATACTCTGGCTTCACAG<br>e2-AR Reverse: CAGATCAGGCAGGTCTTCTG<br>NOVA2 Forward: CCAAGCAGGCCAAGCTGATCGT<br>NOVA2 Reverse: ttaGGCCTTGTGCACCTGCTCG<br>HUB/HUR Forward: ATCGTCAACTACCTCCCTCAGAACATG<br>HUB/HUR Reverse: TGTTGATCGCTCTCTCTGCATCCT<br>hnRNP E1/E2 Forward: GGTCACCCTGAGGCTGGTGG<br>hnRNP E1/E2 Reverse: ATATCCCCTGCCACCTGGACCT<br>hnRNP A2B1 Forward: GAACATCACCTTAGAGATTACTTTGAGGA<br>hnRNP A2B1 Reverse: CTAGACAAAGCCTTTCTTACTTCTGCA<br>GAPDH Forward: ATCACCATCTTCCAGGAGCGAG<br>GAPDH Reverse: GAGATGATGACCCTTTTGGCTCC<br>PSA Forward: GGCCAGGTATTTCAGGTCAG<br>PSA Reverse: TCGTGGCTGGAGTCATCACsiTR3: CAGUCCAGCCAUGCUCCUC(dTdT)
siRNA oligo	siCtrl: ACCCCGGAGAUGCUACCCGAA(dTdT)
single strand RNA	ssRNA oligo: [Biotin]GAUUUGAAAAGAUUUAAUUUCCUCCCUUCU

Table S2. Antibodies and sources.

Antibodies	Sources
Anti-AR-V7 (68492)	Cell signaling
Anti-AR (06-680)	Millipore
Anti-AR (sc-815); Anti-AR (sc-7305), Anti-GAPDH (sc-25778), Anti-GFP (sc-8334); Anti-hnRNP A2B1 (sc-374056), Anti-hnRNP E1/E2 (sc-393076); Anti-HUR (sc-5261); Anti-TR3 (sc-365113); Anti-Nur77 (sc-166166); Rabbit-anti-goat (sc-2768)	Santa Cruz
Anti-FLAG (F7425)	Sigma-Aldrich
Anti-NOVA-2 (ARP40399_T100)	Aviva Systems Biology
Goat anti-Mouse IgG (H+L) Alexa Fluor568 (A-11004); Goat anti-Mouse IgG (H+L), HRP (31430); Goat anti-Rabbit IgG (H+L), HRP (31460)	ThermoFisher Scientific
