# Supplementary material for: TR3 Enhances AR Variant Production and Transactivation, Promoting Androgen Independence of Prostate Cancer Cells
Source: Cancers (Basel). 2022 Apr 10;14(8):1911. doi: 10.3390/cancers14081911 (PMC9031921; doi:10.3390/cancers14081911)
Supplement: Supplementary file 1 [file cancers-14-01911-s001.zip › cancers-1582727-Supporting Information Figure S7.pptx]

## Slide 1
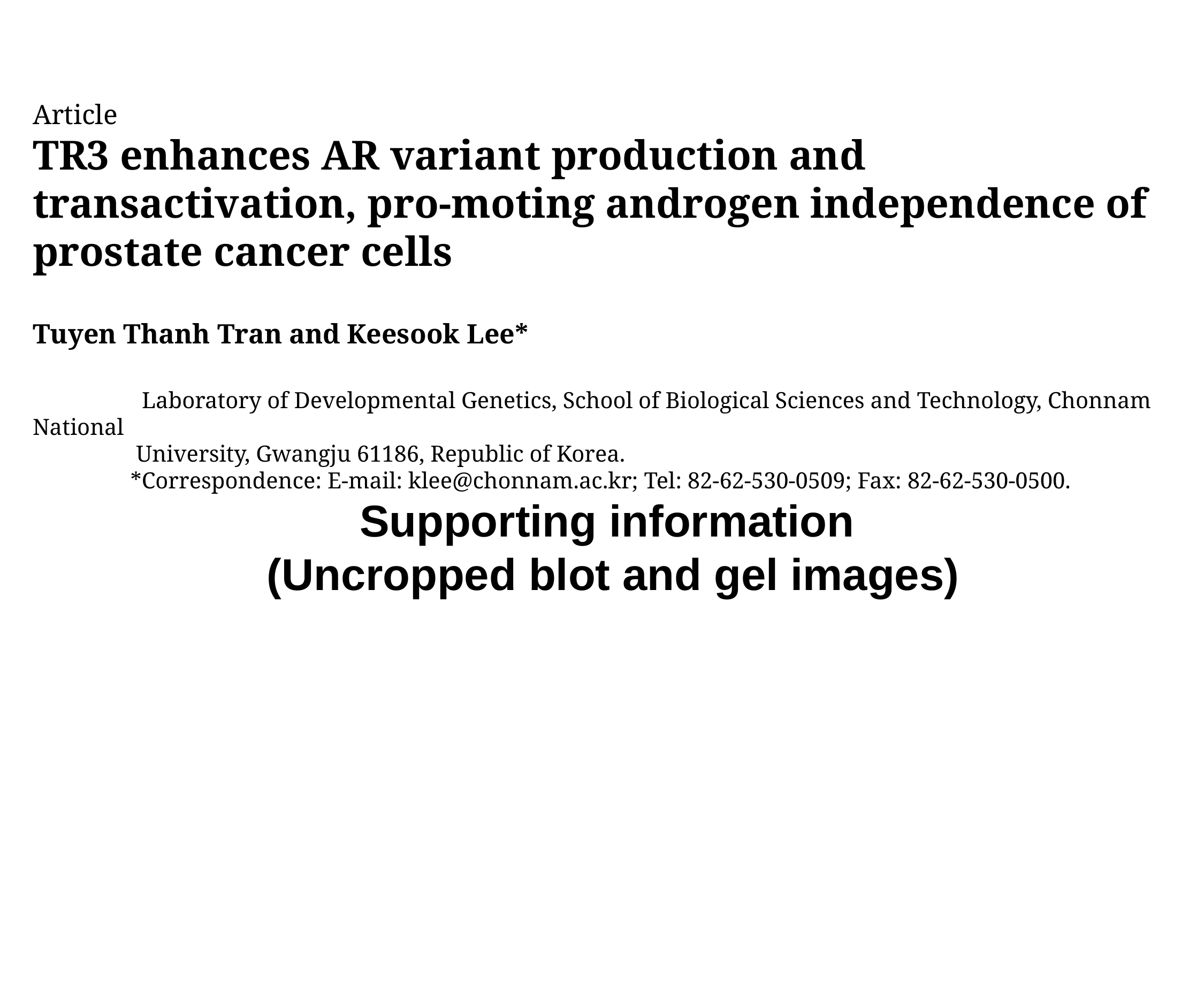

Article
TR3 enhances AR variant production and transactivation, pro-moting androgen independence of prostate cancer cells
Tuyen Thanh Tran and Keesook Lee*
 Laboratory of Developmental Genetics, School of Biological Sciences and Technology, Chonnam National
 University, Gwangju 61186, Republic of Korea.
 *Correspondence: E-mail: klee@chonnam.ac.kr; Tel: 82-62-530-0509; Fax: 82-62-530-0500.
Supporting information
(Uncropped blot and gel images)

## Slide 2
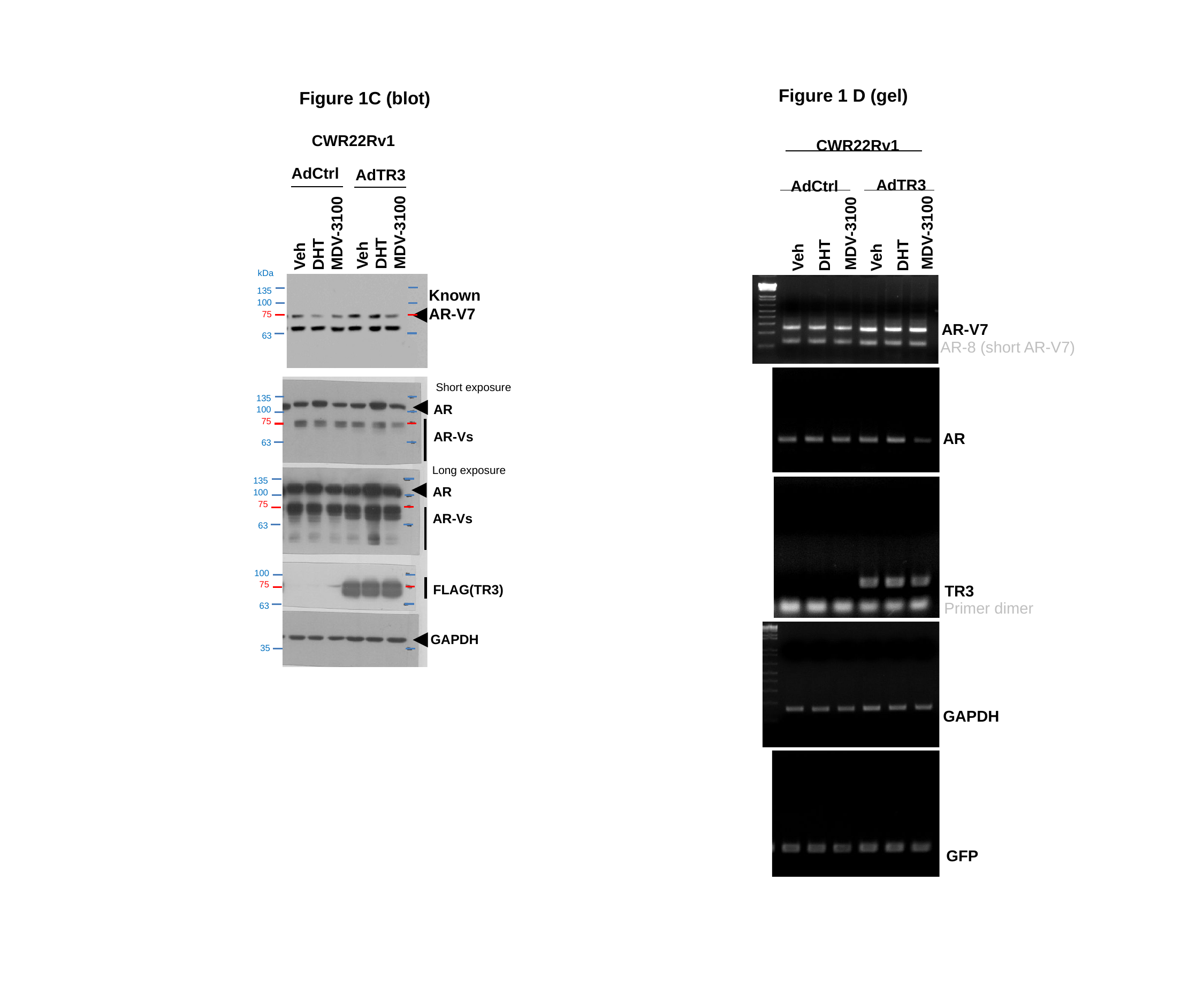

Figure 1 D (gel)
Figure 1C (blot)
CWR22Rv1
AdTR3
AdCtrl
MDV-3100
MDV-3100
Veh
Veh
DHT
DHT
AR-V7
AR-8 (short AR-V7)
AR
TR3
Primer dimer
GAPDH
GFP
CWR22Rv1
AdCtrl
AdTR3
Veh
DHT
MDV-3100
Veh
DHT
MDV-3100
kDa
135
100
75
63
Known
AR-V7
Short exposure
135
100
75
63
AR
AR-Vs
Long exposure
135
100
75
63
AR
AR-Vs
100
75
63
FLAG(TR3)
GAPDH
35

## Slide 3
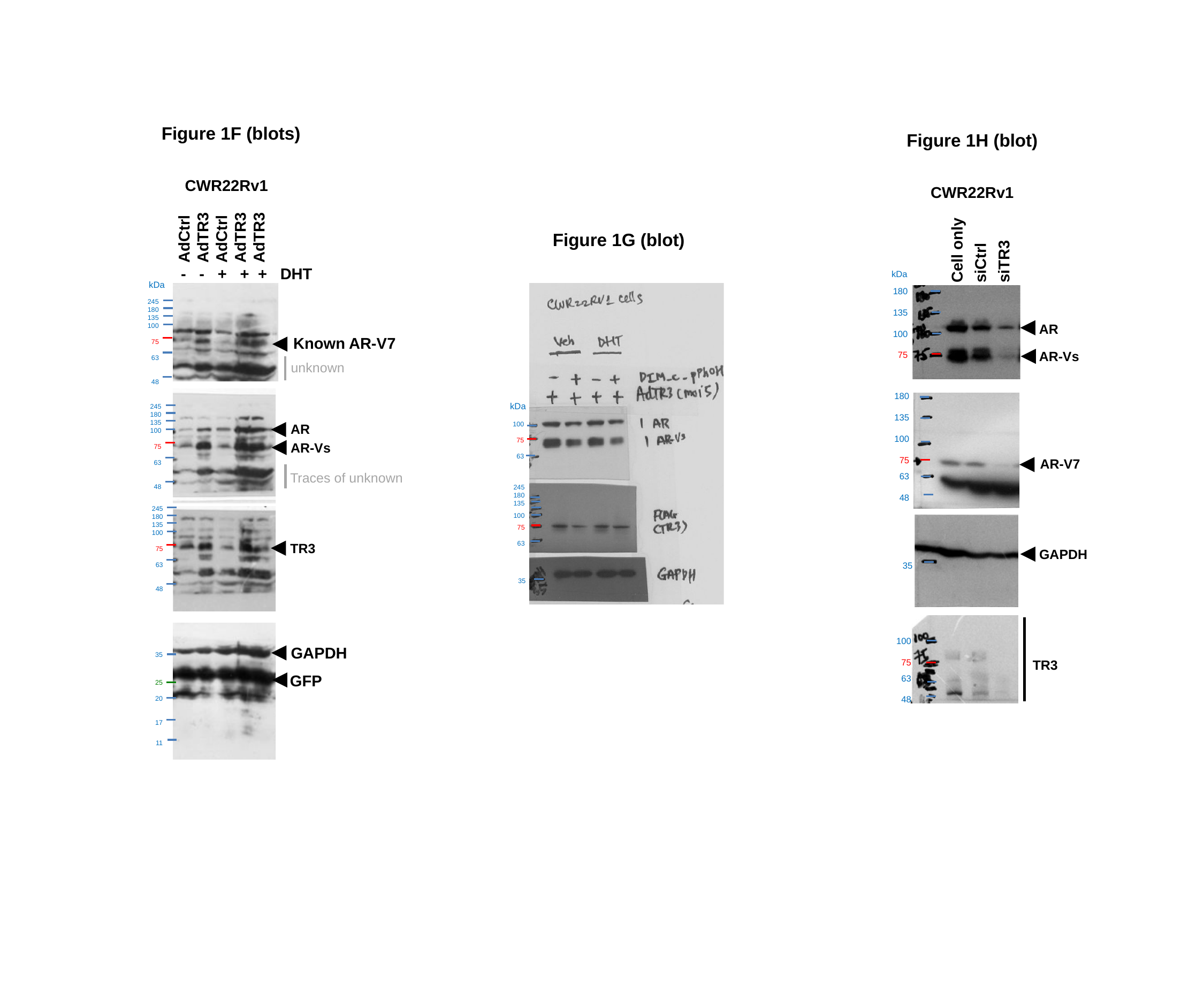

Figure 1H (blot)
CWR22Rv1
Cell only
siCtrl
siTR3
AR
AR-Vs
AR-V7
GAPDH
TR3
kDa
180
135
100
75
180
135
100
75
63
48
35
100
75
63
48
Figure 1F (blots)
AdCtrl
AdTR3
AdCtrl
AdTR3
AdTR3
CWR22Rv1
- - + + + DHT
kDa
245
180
135
100
75
63
48
Known AR-V7
unknown
245
180
135
100
75
63
48
AR
AR-Vs
Traces of unknown
245
180
135
100
75
63
48
TR3
GAPDH
35
25
20
17
11
GFP
Figure 1G (blot)
kDa
100
75
63
245
180
135
100
75
63
35

## Slide 4
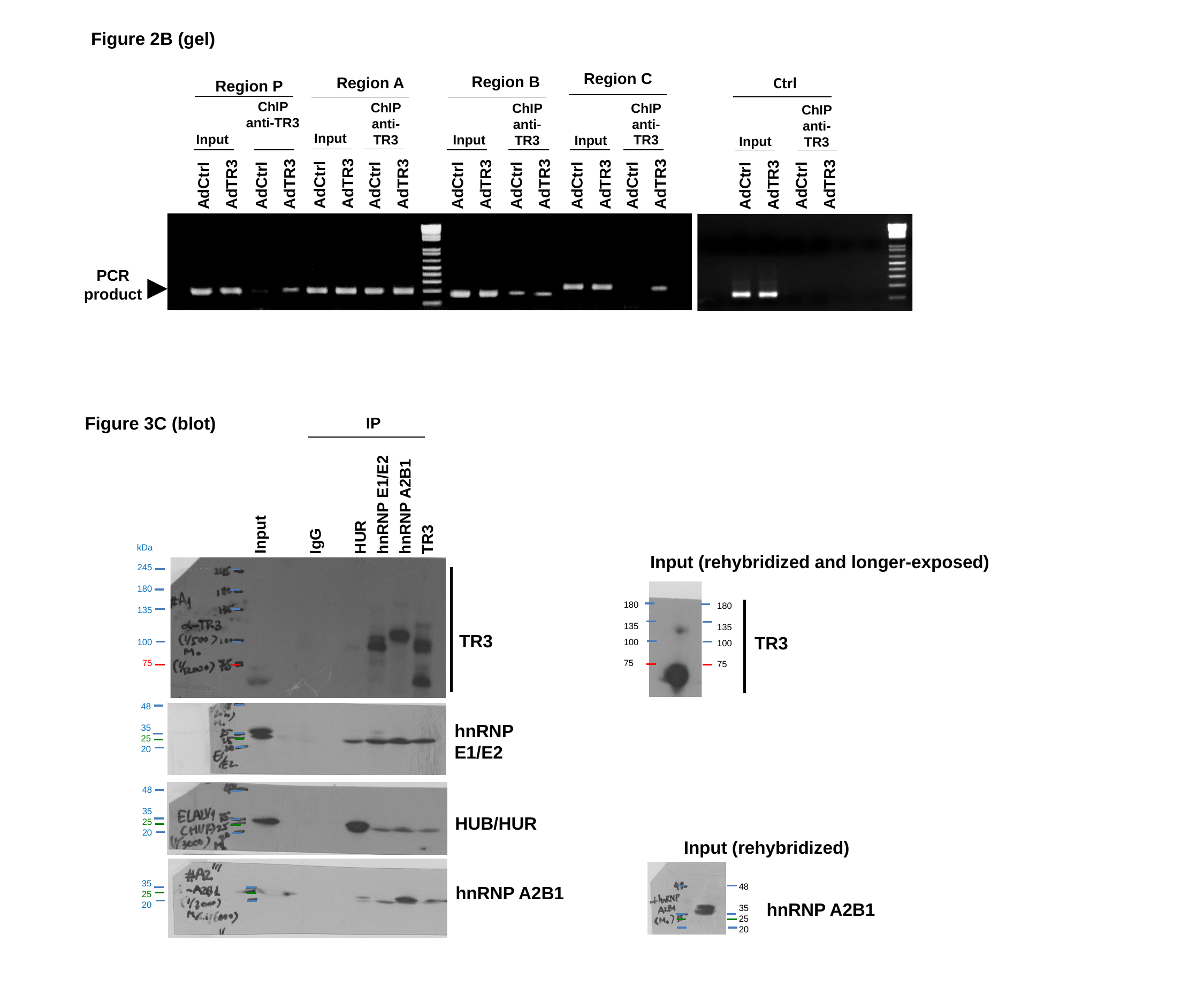

Figure 2B (gel)
Region C
Region B
Region A
Ctrl
Region P
ChIP anti-TR3
ChIP anti-TR3
ChIP anti-TR3
ChIP anti-TR3
ChIP anti-TR3
Input
Input
Input
Input
Input
AdCtrl
AdTR3
AdCtrl
AdTR3
AdCtrl
AdTR3
AdCtrl
AdTR3
AdCtrl
AdTR3
AdCtrl
AdTR3
AdCtrl
AdTR3
AdCtrl
AdTR3
AdCtrl
AdTR3
AdCtrl
AdTR3
PCR product
Figure 3C (blot)
IP
IgG
HUR
hnRNP E1/E2
hnRNP A2B1
TR3
Input
kDa
245
180
135
100
75
TR3
48
35
25
20
hnRNP E1/E2
48
35
25
20
HUB/HUR
35
25
20
hnRNP A2B1
Input (rehybridized and longer-exposed)
180
135
100
75
180
135
100
75
TR3
Input (rehybridized)
48
35
25
20
hnRNP A2B1

## Slide 5
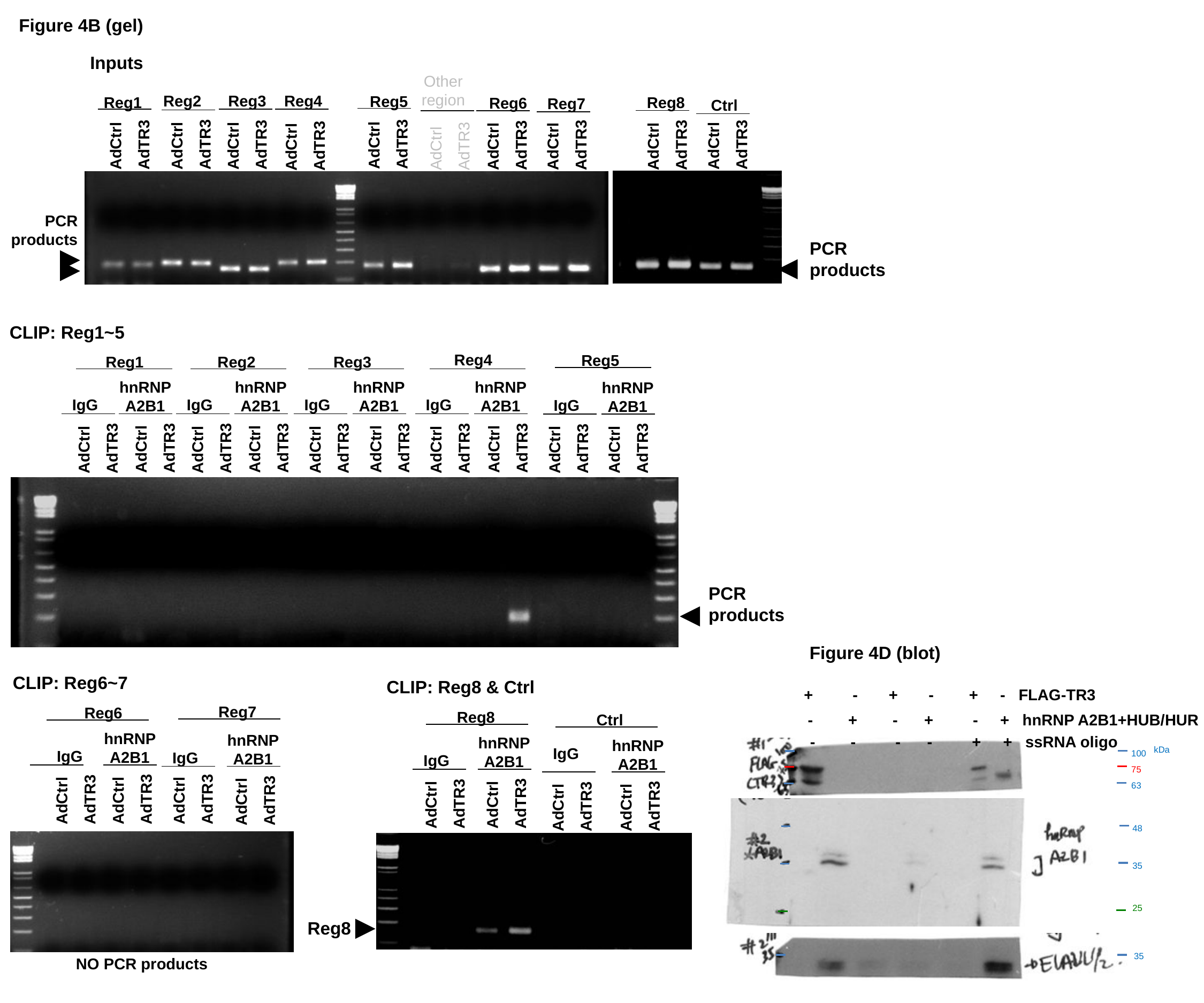

Figure 4B (gel)
Inputs
Other region
Reg2
Reg3
Reg4
Reg5
Reg8
Reg1
Reg6
Reg7
Ctrl
AdCtrl
AdTR3
AdCtrl
AdTR3
AdCtrl
AdTR3
AdCtrl
AdTR3
AdCtrl
AdTR3
AdCtrl
AdTR3
AdCtrl
AdTR3
AdCtrl
AdTR3
AdCtrl
AdTR3
AdCtrl
AdTR3
PCR products
PCR products
CLIP: Reg1~5
Reg4
Reg5
Reg1
Reg2
Reg3
hnRNP A2B1
hnRNP A2B1
hnRNP A2B1
hnRNP A2B1
hnRNP A2B1
IgG
IgG
IgG
IgG
IgG
AdCtrl
AdTR3
AdCtrl
AdTR3
AdCtrl
AdTR3
AdCtrl
AdTR3
AdCtrl
AdTR3
AdCtrl
AdTR3
AdCtrl
AdTR3
AdCtrl
AdTR3
AdCtrl
AdTR3
AdCtrl
AdTR3
PCR products
Figure 4D (blot)
+ - + - + - FLAG-TR3
- + - + - + hnRNP A2B1+HUB/HUR
- - - - + + ssRNA oligo
100
75
63
kDa
48
35
25
35
CLIP: Reg6~7
Reg7
Reg6
hnRNP A2B1
hnRNP A2B1
IgG
IgG
AdCtrl
AdTR3
AdCtrl
AdTR3
AdCtrl
AdTR3
AdCtrl
AdTR3
NO PCR products
CLIP: Reg8 & Ctrl
Reg8
Ctrl
hnRNP A2B1
hnRNP A2B1
IgG
IgG
AdCtrl
AdTR3
AdCtrl
AdTR3
AdCtrl
AdTR3
AdCtrl
AdTR3
Reg8

## Slide 6
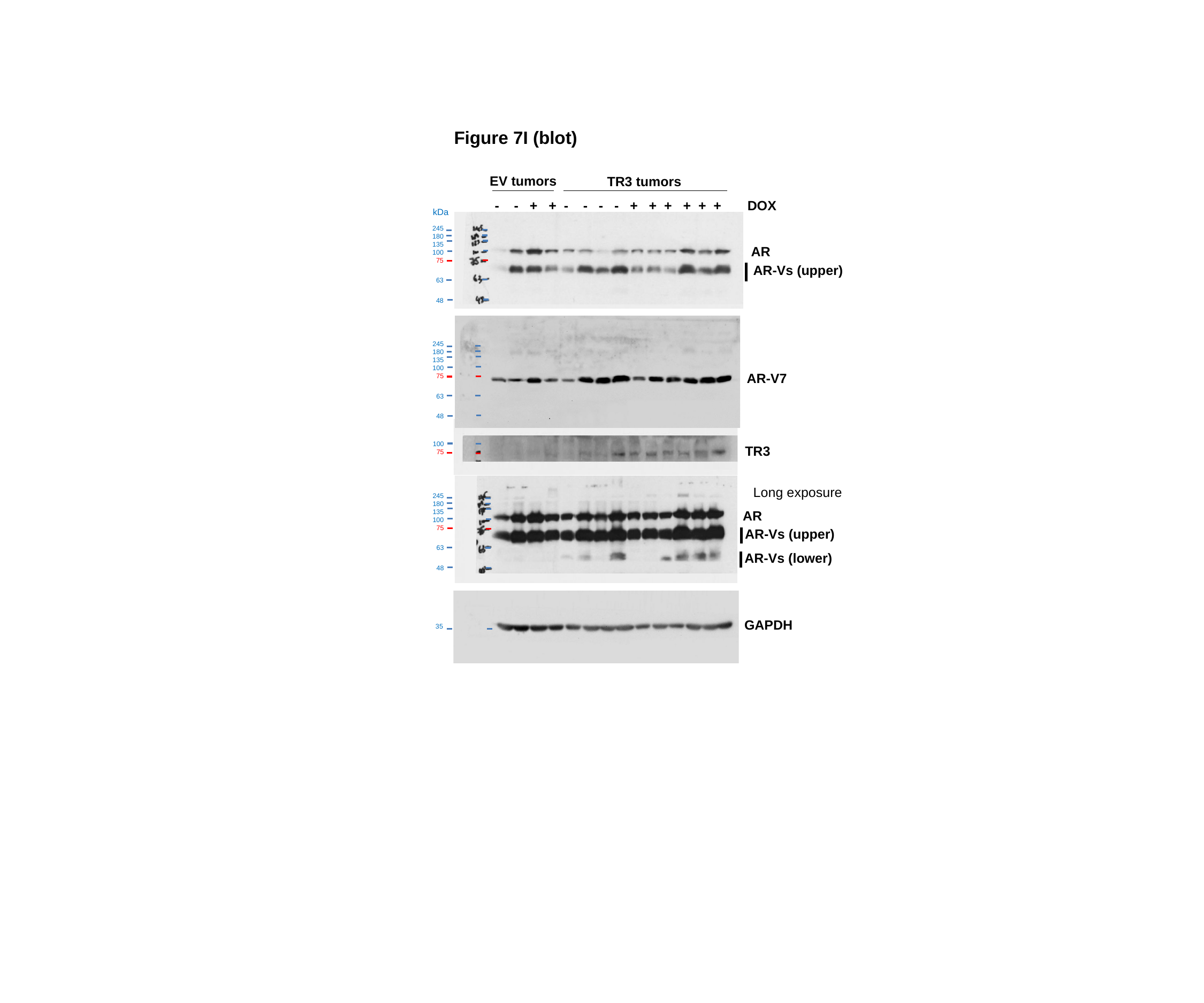

Figure 7I (blot)
EV tumors
TR3 tumors
- - + + - - - - + + + + + + DOX
kDa
245
180
135
100
75
63
48
AR
AR-Vs (upper)
245
180
135
100
75
63
48
AR-V7
100
75
TR3
Long exposure
245
180
135
100
75
63
48
AR
AR-Vs (upper)
AR-Vs (lower)
35
GAPDH

## Slide 7
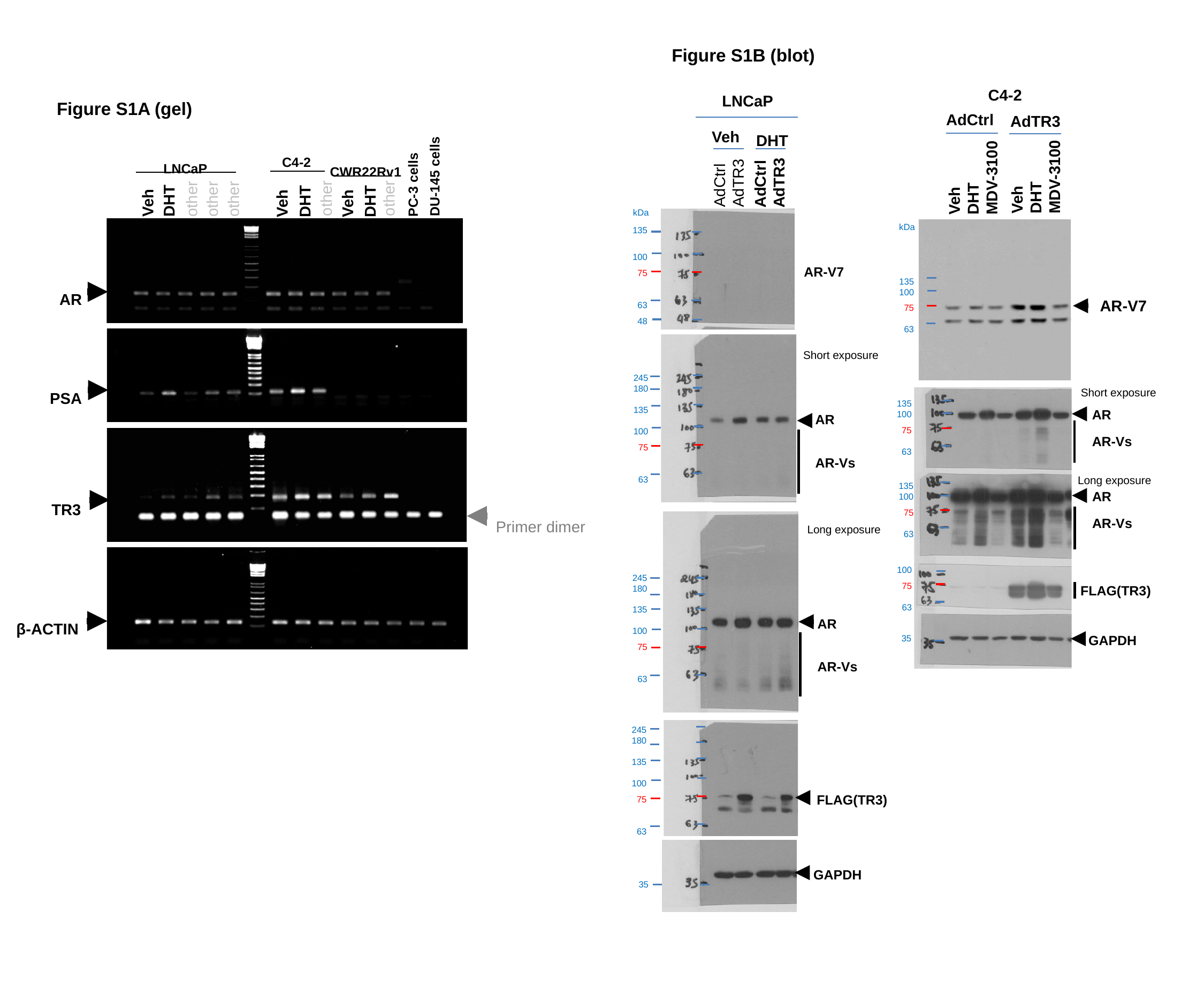

Figure S1B (blot)
C4-2
AdCtrl
AdTR3
Veh
DHT
MDV-3100
Veh
DHT
MDV-3100
kDa
135
100
75
63
AR-V7
Short exposure
135
100
75
63
AR
AR-Vs
135
100
75
63
Long exposure
AR
AR-Vs
100
75
63
FLAG(TR3)
GAPDH
35
C4-2
LNCaP
CWR22Rv1
DU-145 cells
PC-3 cells
other
other
other
other
other
Veh
DHT
Veh
DHT
Veh
DHT
AR
PSA
TR3
Primer dimer
β-ACTIN
LNCaP
Veh
DHT
AdCtrl
AdTR3
AdCtrl
AdTR3
kDa
135
100
75
63
48
AR-V7
Short exposure
245
180
135
100
75
63
AR
AR-Vs
Long exposure
245
180
135
100
75
63
AR
AR-Vs
245
180
135
100
75
63
FLAG(TR3)
GAPDH
35
Figure S1A (gel)

## Slide 8
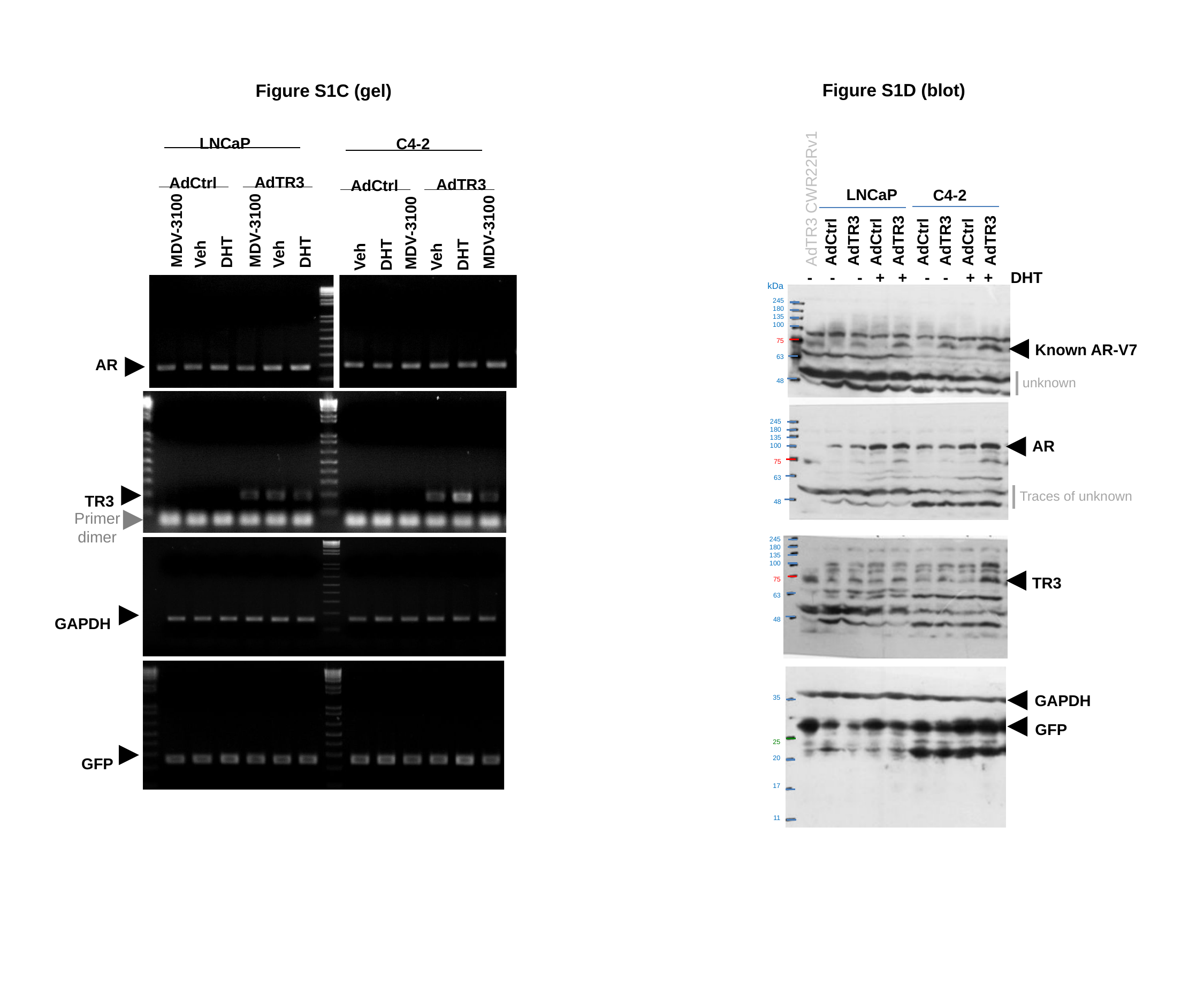

Figure S1D (blot)
Figure S1C (gel)
LNCaP
C4-2
AdTR3
AdCtrl
AdTR3
AdCtrl
MDV-3100
MDV-3100
Veh
Veh
MDV-3100
MDV-3100
Veh
Veh
DHT
DHT
DHT
DHT
AR
TR3
Primer dimer
GAPDH
GFP
AdCtrl
AdTR3
AdCtrl
AdTR3
AdCtrl
AdTR3
AdCtrl
AdTR3
AdTR3 CWR22Rv1
LNCaP
C4-2
- - - + + - - + + DHT
kDa
245
180
135
100
75
63
48
Known AR-V7
unknown
245
180
135
100
75
63
48
AR
Traces of unknown
245
180
135
100
75
63
48
TR3
GAPDH
35
25
20
17
11
GFP

## Slide 9
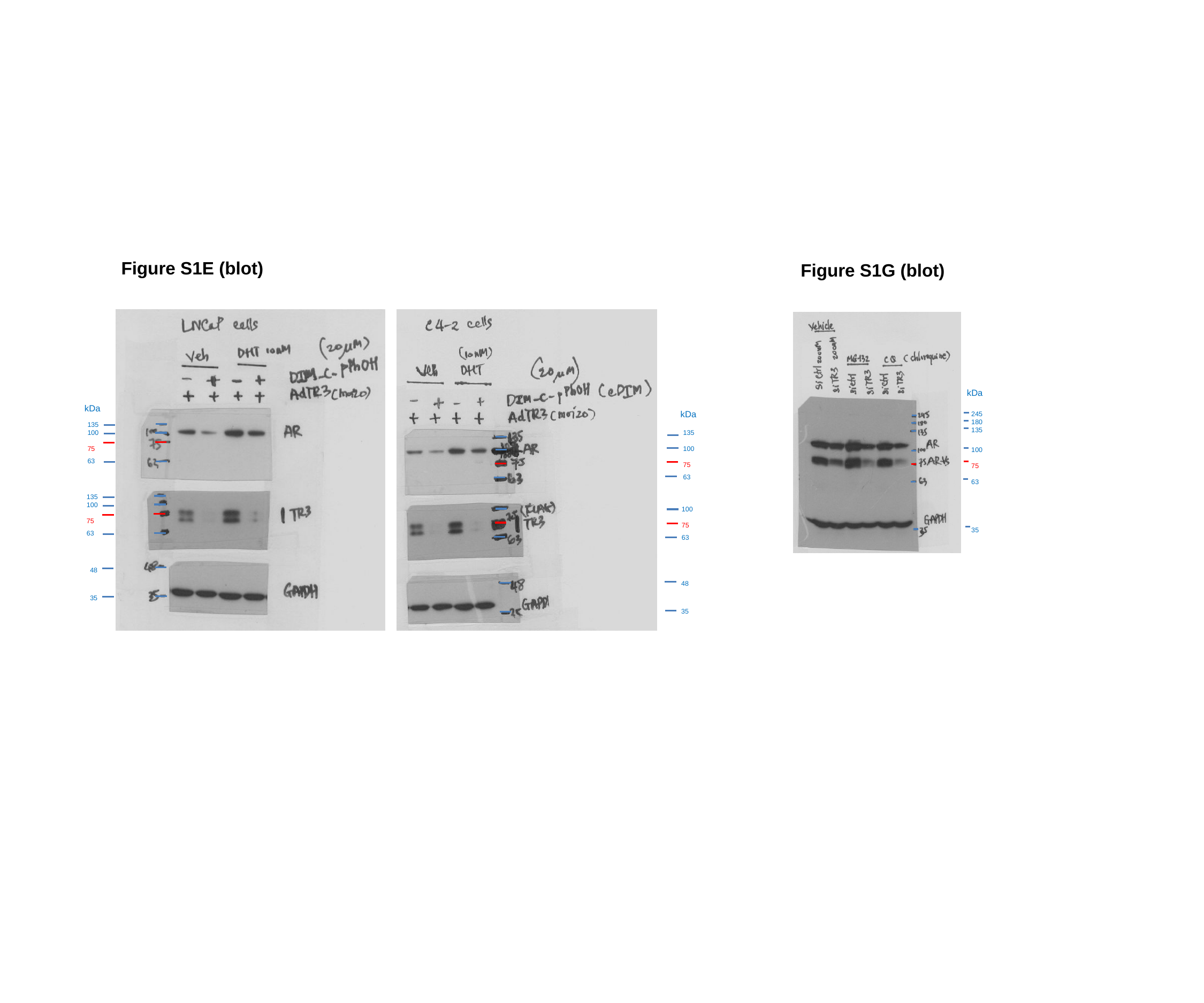

Figure S1E (blot)
Figure S1G (blot)
kDa
135
100
75
63
135
100
75
63
48
35
kDa
135
100
75
63
100
75
63
48
35
kDa
245
180
135
100
75
63
35

## Slide 10
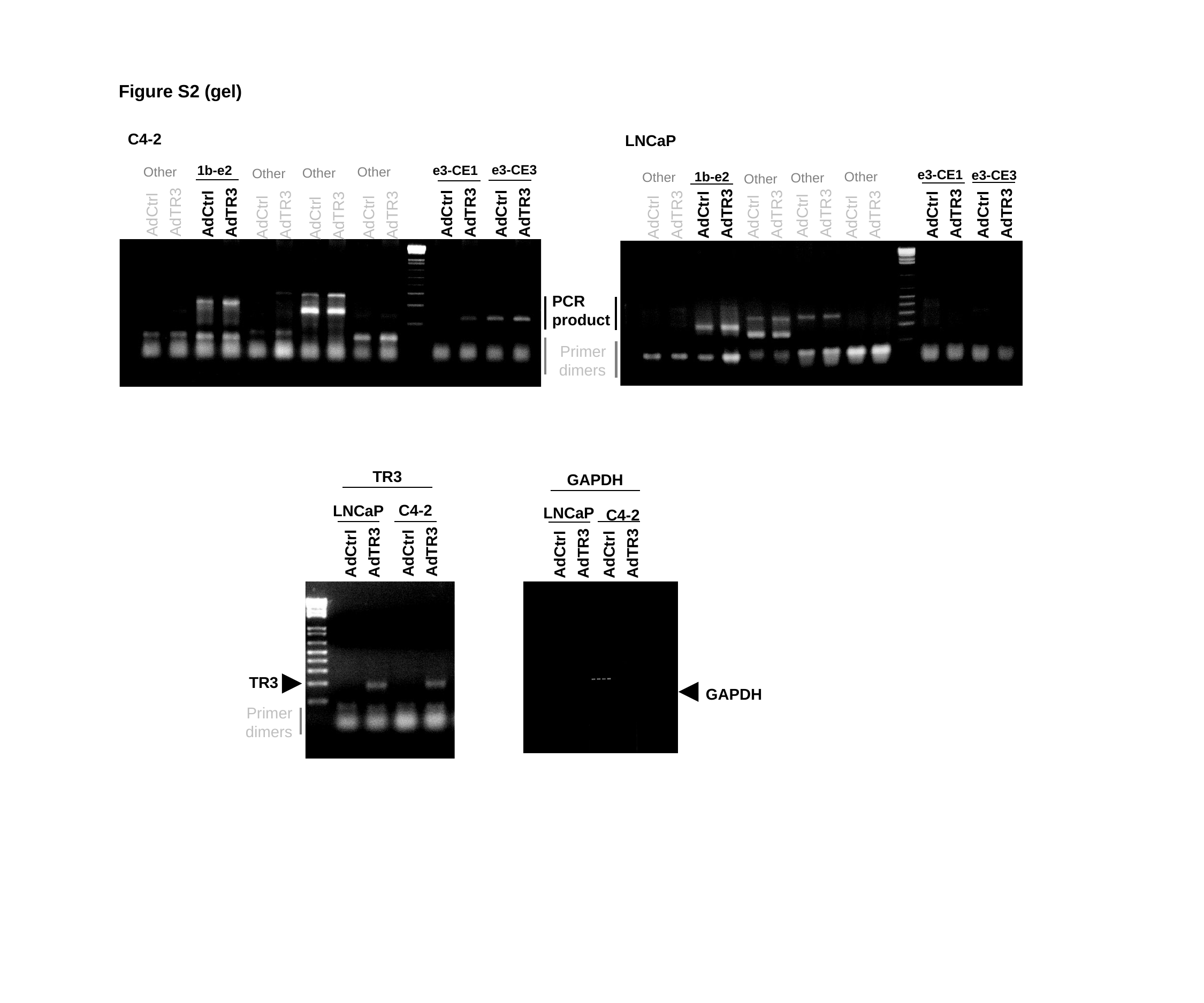

Figure S2 (gel)
C4-2
e3-CE3
1b-e2
e3-CE1
Other
Other
Other
Other
AdCtrl
AdTR3
AdCtrl
AdTR3
AdCtrl
AdTR3
AdCtrl
AdTR3
AdCtrl
AdTR3
AdCtrl
AdTR3
AdCtrl
AdTR3
LNCaP
e3-CE1
e3-CE3
1b-e2
Other
Other
Other
Other
AdCtrl
AdTR3
AdCtrl
AdTR3
AdCtrl
AdTR3
AdCtrl
AdTR3
AdCtrl
AdTR3
AdCtrl
AdTR3
AdCtrl
AdTR3
PCR
product
Primer dimers
TR3
C4-2
LNCaP
AdCtrl
AdTR3
AdCtrl
AdTR3
TR3
Primer dimers
GAPDH
LNCaP
C4-2
AdCtrl
AdTR3
AdCtrl
AdTR3
GAPDH

## Slide 11
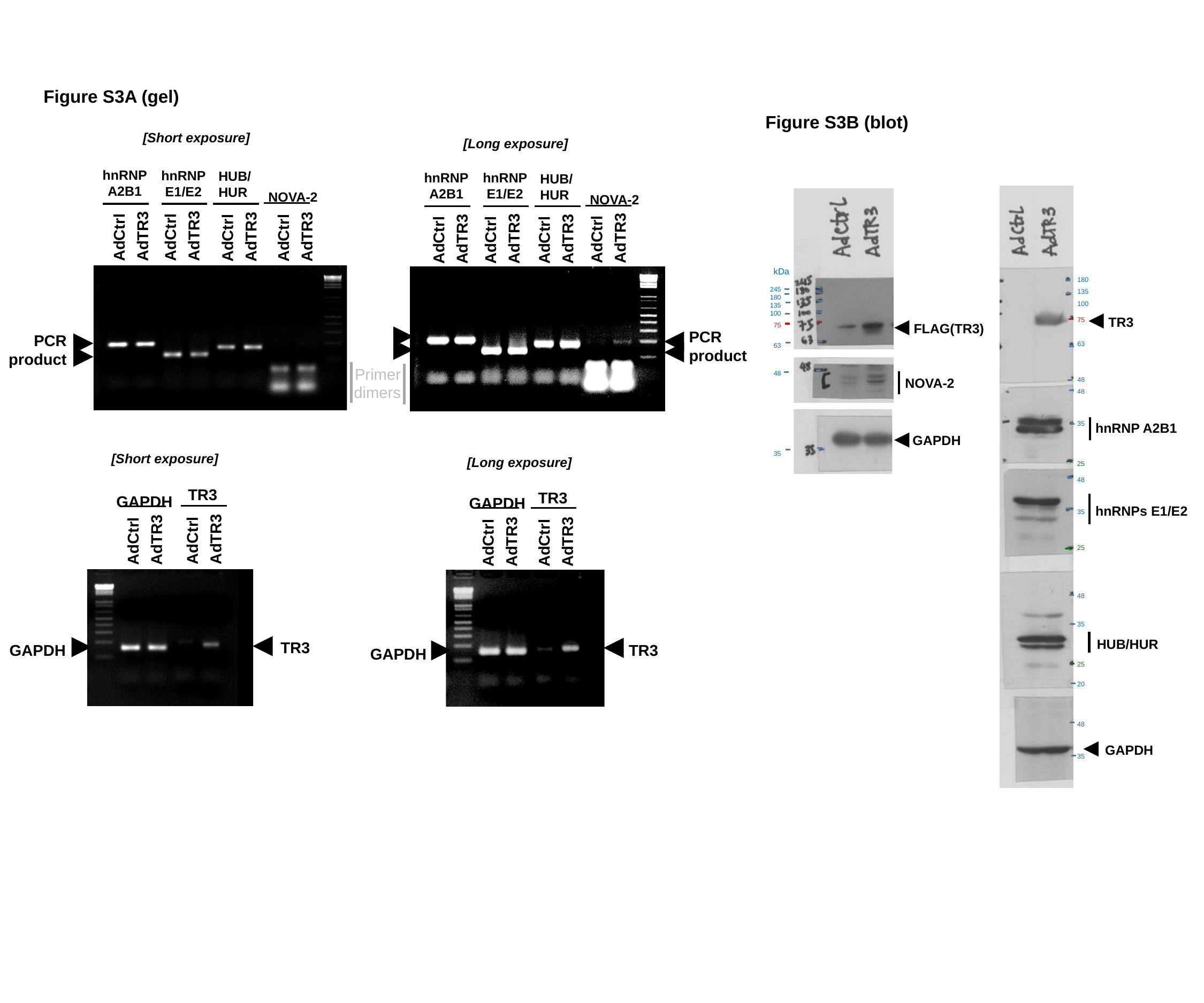

Figure S3A (gel)
Figure S3B (blot)
[Short exposure]
hnRNP
A2B1
hnRNP
E1/E2
HUB/HUR
NOVA-2
AdCtrl
AdTR3
AdCtrl
AdTR3
AdCtrl
AdTR3
AdCtrl
AdTR3
PCR product
[Long exposure]
hnRNP
A2B1
hnRNP
E1/E2
HUB/HUR
NOVA-2
AdCtrl
AdTR3
AdCtrl
AdTR3
AdCtrl
AdTR3
AdCtrl
AdTR3
PCR product
180
135
100
75
63
48
TR3
48
35
25
48
35
25
48
35
25
20
48
35
hnRNP A2B1
hnRNPs E1/E2
HUB/HUR
GAPDH
kDa
245
180
135
100
75
63
48
35
FLAG(TR3)
NOVA-2
GAPDH
Primer dimers
[Short exposure]
GAPDH
TR3
AdCtrl
AdTR3
AdCtrl
AdTR3
GAPDH
TR3
[Long exposure]
GAPDH
TR3
AdCtrl
AdTR3
AdCtrl
AdTR3
GAPDH
TR3

## Slide 12
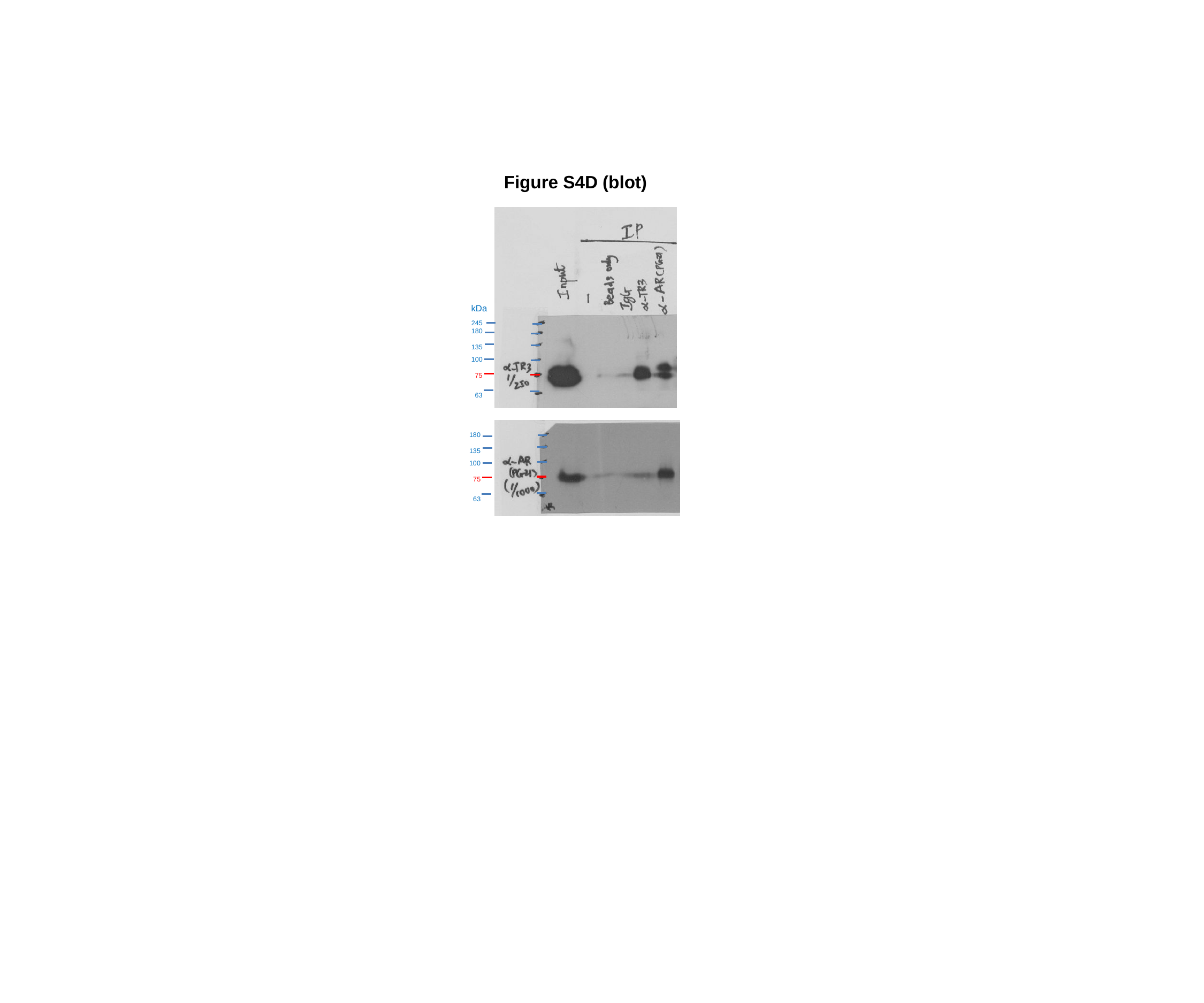

Figure S4D (blot)
kDa
245
180
135
100
75
63
180
135
100
75
63
